# Intraspecific Relationships among Wood Density, Leaf Structural Traits and Environment in Four Co-Occurring Species of *Nothofagus* in New Zealand

**DOI:** 10.1371/journal.pone.0058878

**Published:** 2013-03-18

**Authors:** Sarah J. Richardson, Robert B. Allen, Rowan P. Buxton, Tomás A. Easdale, Jennifer M. Hurst, Christopher W. Morse, Rob D. Smissen, Duane A. Peltzer

**Affiliations:** Landcare Research, Lincoln, New Zealand; Michigan State University, United States of America

## Abstract

Plant functional traits capture important variation in plant strategy and function. Recent literature has revealed that within-species variation in traits is greater than previously supposed. However, we still have a poor understanding of how intraspecific variation is coordinated among different traits, and how it is driven by environment. We quantified intraspecific variation in wood density and five leaf traits underpinning the leaf economics spectrum (leaf dry matter content, leaf mass per unit area, size, thickness and density) within and among four widespread *Nothofagus* tree species in southern New Zealand. We tested whether intraspecific relationships between wood density and leaf traits followed widely reported interspecific relationships, and whether variation in these traits was coordinated through shared responses to environmental factors. Sample sites varied widely in environmental variables, including soil fertility (25–900 mg kg^–1^ total P), precipitation (668–4875 mm yr^–1^), temperature (5.2–12.4 °C mean annual temperature) and latitude (41–46 °S). Leaf traits were strongly correlated with one another within species, but not with wood density. There was some evidence for a positive relationship between wood density and leaf tissue density and dry matter content, but no evidence that leaf mass or leaf size were correlated with wood density; this highlights that leaf mass per unit area cannot be used as a surrogate for component leaf traits such as tissue density. Trait variation was predicted by environmental factors, but not consistently among different traits; e.g., only leaf thickness and leaf density responded to the same environmental cues as wood density. We conclude that although intraspecific variation in wood density and leaf traits is strongly driven by environmental factors, these responses are not strongly coordinated among functional traits even across co-occurring, closely-related plant species.

## Introduction

Consistent correlations among plant traits, and between plant traits and environment, are the basis for defining and interpreting plant strategies [1]–[3]. Interspecific correlations among leaf traits and their relationships with the environment have been thoroughly documented [4]–[6] especially those contributing to the ‘leaf economics spectrum’ [2]. Although there is clear evidence for consistent trait correlations and trait syndromes *among* species, these have not been rigorously tested *within* species. Recent studies have revealed that intraspecific variation is a major component of trait variation, both within and among communities, more so than previously supposed [7]–[12]. Intraspecific and intrageneric studies are a strong test of the functional link between traits [13], [14] because they avoid the problem of trait associations derived from shared ancestry and fortuitous trait divergences [15]. Further, as different mechanisms may constrain trait–trait combinations within species to those widely observed among species [16], [17], studies of both inter- and intraspecific variation are valuable for gaining a comprehensive view of the drivers of trait variation. Similarly, intraspecific studies of trait–environment relationships complement studies of trait variation among communities as these are confounded by species turnover along environmental gradients [18]–[20]. Intraspecific correlations among plant traits and with environment have great promise for revealing functional associations among traits, and between traits and the environment [19], [21].

Leaf mass per unit area (LMA) forms the backbone of the leaf economics spectrum because it integrates several aspects of leaf construction including leaf dry matter content (LDMC), tissue density and leaf thickness [4], [5], [22]. LMA is also closely linked to leaf-level processes such as photosynthetic rate, as well as whole-plant performance [23]. Although LMA and its component traits are typically intercorrelated, the individual components of LMA can also vary somewhat independently, reflecting their specific functions and responses to the environment [4], [24]–[26]. LMA is strongly linked to photosynthetic rates through the influence of leaf thickness and leaf density on leaf volume and intercellular resistance to CO_2_ conductance, respectively [4]. LDMC and leaf density have been advocated as better predictors of plant performance along resource gradients than LMA, because they are more direct measures of leaf construction and allocation [5], [24], [26]. Given the unique functions of each component trait, we propose that the relationships between each leaf trait and other plant functional traits (e.g., stem and root traits) cannot be generalised from LMA alone. Further, leaf trait responses to environment are likely to vary according to the specific function of each component trait of LMA. In this study, we test the consistency of relationships between leaf traits and wood density – a key stem trait – within species using several component traits of the leaf economic spectrum rather than solely LMA.

Interspecific leaf trait variation has been linked to variation in roots [15], [27] and stems [28] in an attempt to define whole-plant axes of trait variation [3]. Wood density is one of the more widely studied stem traits [29], [30] as it is an easily measured estimate of resource allocation to structural support. Despite the expectation that leaf and wood traits should be closely coupled along a spectrum of plant strategies from fast-growing, resource-acquiring species with low investment in leaf and wood tissues to slow-growing, resource-conserving species with high investment [28], [29], relationships between wood density and leaf traits are complex and often point to orthogonal rather than parallel axes of variation. However, many studies report that species having dense wood also have relatively small leaves [31]–[34]. This has been interpreted as an adaptation to water limitation, with smaller leaves and higher wood density both promoting greater water use efficiency [35], [36]. Hence, traits are correlated through a shared link to an environmental factor. Shared responses to environment drive correlations between traits, leading to shifts in trait syndromes along environmental gradients. High LMA is sometimes associated with high wood density [37]–[39], but non-significant relationships have also been reported [36], [40], [41]. Correlations between wood density and the component traits underpinning LMA (i.e., LDMC, leaf size, leaf thickness and leaf density) have received relatively little attention but indicate that species with a high wood density also have high LDMC [37], [42] while relationships with leaf density and thickness are less clear.

The two studies linking intraspecific variation in wood density and LMA have reached contradictory conclusions. There was no relationship between LMA and wood density for *Pinus sylvestris* sampled across Europe [19], whereas LMA and wood density of *Nothofagus pumilio* were correlated across sites, but not within individual sites along elevation gradients [21]. Intraspecific relationships between wood density and other component leaf traits of LMA have not been investigated either within communities or across wide environmental gradients.

We quantified intraspecific variation in wood density and five leaf traits that underpin the leaf economics spectrum within four species of *Nothofagus* throughout the South Island of New Zealand. Our sampling quantified intraspecific trait variation of *Nothofagus* species both within and among 30 sites, and encompassed wide environmental variation along both latitudinal and altitudinal gradients. *Nothofagus* is the most abundant tree genus in New Zealand forests, is common throughout the Southern Hemisphere and is ideal for evaluating the extent of intraspecific variation under natural conditions at large (i.e., >700 km) spatial scales. We first test whether LMA and the component leaf traits contributing to LMA show consistent relationships with wood density within each species. We specifically test whether the widely reported interspecific relationships between wood density and leaf traits are upheld within species. We then examine whether wood density and each of the five leaf traits share common responses to environmental variation. Finally, these data are used to evaluate whether LMA is a functional surrogate for the underpinning component traits, or whether each leaf trait exhibited distinctive functional responses to variation in wood density and the environment.

## Methods

### Site and species selection

**Figure 1:**
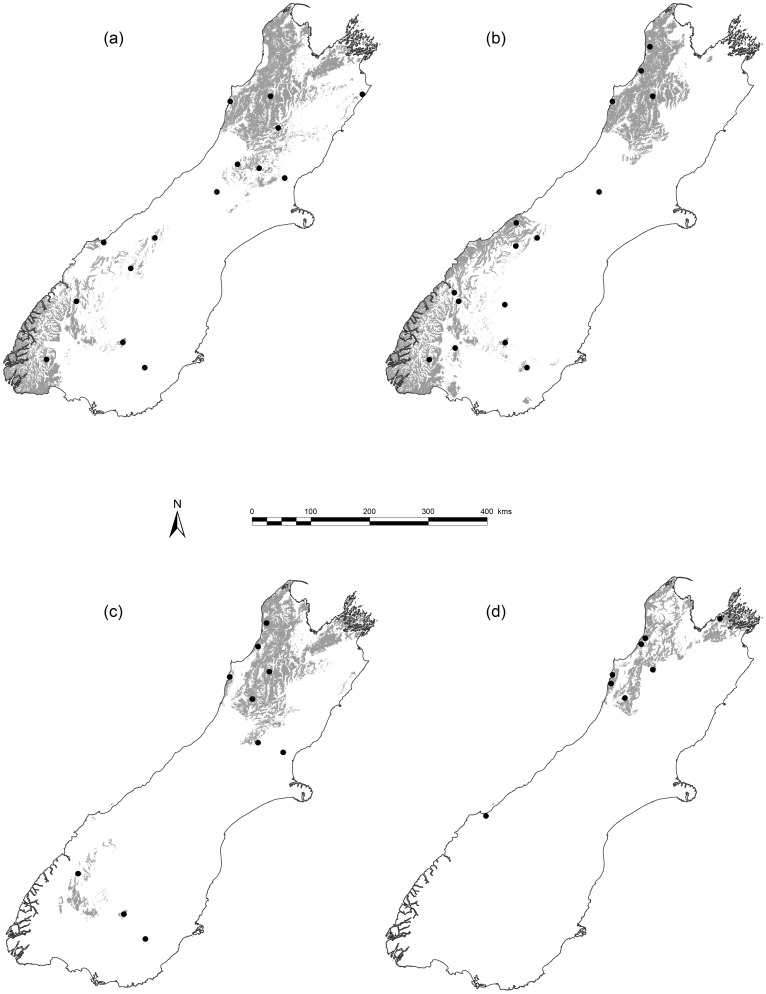
Distribution of four *Nothofagus* spp. in New Zealand and sampling locations for six functional traits. Sampling locations (filled circles) are shown relative to modelled distributions [71] in grey shading for (a) *N. solandri* (b) *N. menziesii* (c) *N. fusca* and (d) *N. truncata*.

We sampled the four evergreen *Nothofagus* species found in New Zealand – *N. menziesii* (Hook.f.) Oerst., *N. fusca* (Hook.f.) Oerst., *N. solandri* (Hook.f.) Oerst. and *N. truncata* (Colenso) Cockayne. *N. solandri* is treated here as a single species without distinction between its two commonly recognised varieties (*N. solandri* var. *solandri* and *N. solandri* var. *cliffortioides* (Hook.f.) Poole), as these hybridise abundantly [43] and many intermediate forms were sampled here. All species are common and widespread [44], long-lived (250–600 years), and often dominate forest communities [45]. *N. truncata* has the narrowest environmental niche, being confined to low fertility soils and sites with a mean annual temperature (MAT) of ≥ *c*. 9 °C [45], [46]. *N. fusca* is widespread and excluded only from the coolest (< *c*. 6.5 °C) and driest (< *c*. 600 mm mean annual rainfall, MAR) sites. *N*. *menziesii* and *N*. *solandri* have wide environmental niches and occur from sea level to treeline (MAT *c*. 5 °C) [45], [46]. We sampled *Nothofagus* at 30 sites throughout the environmental diversity of southern New Zealand (Fig. 1; Table S1). Sites were selected to capture the full range of environments encountered by each *Nothofagus* species. Sites were visited during March–April 2010 (late austral summer) to sample stem wood, canopy leaves and mineral soil. We sampled 9–11 canopy trees of each *Nothofagus* species at each site, ranging in diameter at breast height from 51 to 785 mm. Sampled areas were <1000 m^2^ (*c*. 30×30 m) and were selected to be homogeneous, with minimal local variation in soil fertility due to microtopography.

### Plant traits

All individuals sampled were canopy trees with fully illuminated foliage. All trait measurements followed standard protocols [47]. For foliar traits we used 8–12 leaves from each individual of each species at each site to measure: leaf dry matter content (LDMC, mg g^–1^) as the ratio of oven-dried (at 60 °C for 48 h) leaf mass to fully saturated leaf mass (leaves were removed from branches, placed between moist tissue paper and stored in sealed plastic bags in a fridge overnight); leaf thickness (mm) of the lamina (avoiding the midrib) measured using a Measumax digital micrometer; fresh leaf area (mm^2^), using Winfolia and an Epson flatbed scanner (Epson Corporation, Nagano, Japan); leaf mass per unit area (LMA, g m^–2^) as the ratio of dry mass to fresh leaf area; and leaf density (mg mm^–3^) as the ratio of dry mass to fresh leaf volume (fresh leaf area × thickness). Canopy leaves were sampled using a shotgun. We used the mean of the 8–12 leaves from each individual in all analyses. One wood density (oven-dry mass divided by green volume, kg m^–3^) sample was taken at breast height (1.35 m). For stems with dbh ≥ 120 mm, we used a 21-mm auger to sample each individual through to the stem centre. The depth of the augured hole was measured to the nearest millimetre and the volume of the hole calculated as a cylinder. Wood density of stems with dbh of 51–120 mm was measured on stem discs that were kept moist prior to cutting a regular volume in the laboratory using a bandsaw. All wood samples were air-dried (up to 2 months) and then oven-dried (70 °C) over several weeks until a stable mass was obtained. A small number of stems of two species in the dbh range of 80 to 120 mm were measured using the auger and these were compared to samples collected using discs to assess whether the sampling method affected estimates of wood density, while accounting for stem dbh. There was no statistical evidence that sampling method affected wood density in *N. menziesii* (General Linear Model, *F*_1,8_ = 0.01, *P* = 0.948) or *N. solandri* (General Linear Model, *F*_1,13_ = 2.32, *P* = 0.152).

### Climate and soil chemistry

MAT and MAR for each site were modelled using thin-plate splines fitted to data from nearby meteorological stations [48]. Elevation was taken from a topographic map. Soil nutrient availability (total P) was estimated from a pooled mineral soil sample at each site. Five mineral soil (0–100 mm) subsamples were collected systematically from each site with a corer of 65-mm internal diameter. These subsamples were pooled per site and air-dried, sieved through 2-mm mesh, and used for measurement of gravimetric soil water content and total P (ignition and dissolution in 0.5 M sulphuric acid). Results are expressed on an oven-dry-soil (105 °C) basis. Soil total P is used as a single measure of soil nutrient availability as it has demonstrated relationships in New Zealand with forest community composition [49], plant nutrient use efficiency [50] and functional trait spectra [15].

### Statistical analyses

Plant traits can vary with ontogeny [51] and to remove size-related variation in each trait before testing for trait-trait and trait-environment correlations, we used a general linear model to determine the relationship between tree diameter at breast height and each trait within each species. The residuals from these models were used in all subsequent analyses. Pearson's correlations were run for each trait-trait and trait-environment relationship within each species. Statistical significance of each correlation was assessed after Holm-Bonferroni correction tests for multiple comparisons. All traits were log-transformed prior to analysis, to meet assumptions of normality and to linearise trait–trait and trait–environment relationships. In addition to MAT, MAR and soil P, we included elevation and latitude as indirect measures of environmental variation as they commonly emerge as strong predictors of trait variation and are widely-explored ecological gradients [20], [23], [52]. Given that our five environmental predictors are necessarily correlated to some extent (Table S2), we used correlations of each environmental variable and each trait within species to identify consistent signals across species and traits. Additionally, we used multiple regression to predict intraspecific variation in each trait from the five environmental variables, for each species. The goal was to identify whether consistent combinations of environmental variables predicted intraspecific variation in each trait in each of the four species. We used linear regression models and backwards selection to remove non-significant terms from a full model using all five environmental variables.

## Results

The amount of intraspecific variation differed widely across the six plant traits (Table S3). LDMC and wood density both varied less than two-fold and values were conserved within species, resulting in low coefficients of variation (mean CVs across species of 5.2% for LDMC and 8.6% for wood density; Table S3). Leaf thickness, leaf density and LMA varied around two-fold within species, with mean CVs across species of 14.0%, 13.7% and 16.1%, respectively. Leaf size varied widely within species with a mean coefficient of variation across the four species of 30.0% (Table S3).

### Intraspecific correlations between wood density and leaf traits

**Figure 2:**
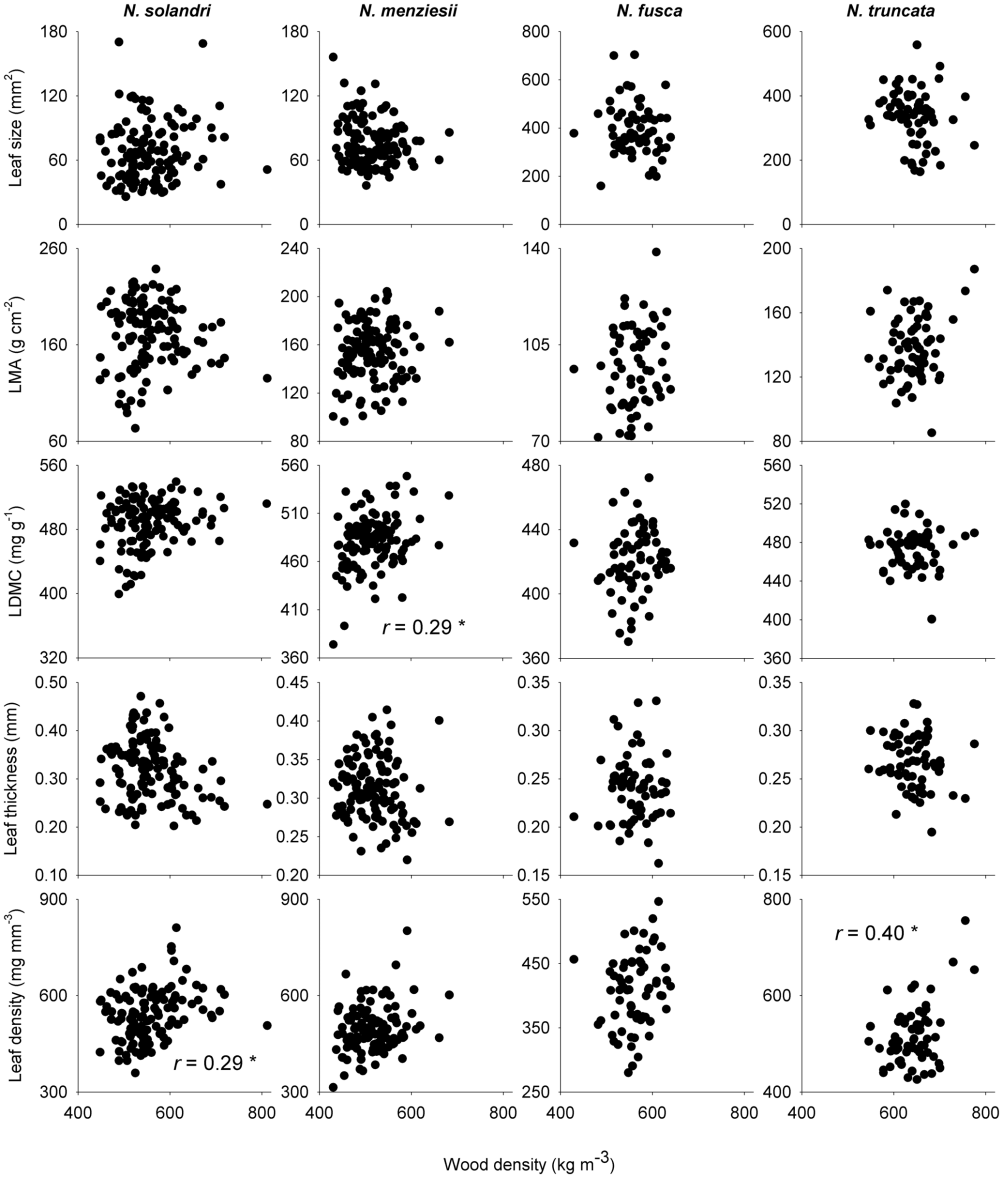
*Nothofagus* spp. wood density and leaf trait relationships in New Zealand. Relationships between wood density and five leaf structural traits are given for four species of *Nothofagus* in New Zealand. See Table 1 for correlation tests. LDMC  =  leaf dry matter content; LMA  =  leaf mass per unit area.

Leaf traits were only correlated with wood density in some species, and for some traits (Table 1; Fig. 2). Leaf size did not decrease with increasing wood density within any of the four species, despite this being the most consistently reported pattern of interspecific covariation between wood density and a leaf trait (Table 1; Fig. 2). Similarly, there was no evidence for an intraspecific relationship between wood density and LMA. There was weak intraspecific evidence that LDMC and leaf density both increased with wood density, pointing to coordinated investment in dry matter allocation to both leaves and wood within species.

**Table 1:** Intraspecific variation in wood density and five component leaf traits for four *Nothofagus* species.

Trait 1	Trait 2	*N. solandri* (*n* = 127)	*N. menziesii* (*n* = 119)	*N. fusca* (*n* = 65)	*N. truncate* (*n* = 65)
Wood density	Leaf size	–0.03	–0.08	–0.11	–0.20
	LMA	0.00	0.07	0.12	0.18
	LDMC	0.22	**0.29**	0.10	0.02
	Leaf thickness	–0.22	–0.13	–0.03	–0.17
	Leaf density	**0.29**	0.22	0.16	**0.40**
					
LMA	LDMC	**0.80**	**0.68**	**0.77**	**0.73**
	Leaf size	**–0.60**	**–0.43**	–0.24	–0.17
	Leaf density	**0.50**	**0.61**	**0.53**	**0.72**
	Leaf thickness	**0.76**	**0.58**	**0.50**	**0.65**
					
LDMC	Leaf size	**–0.65**	**–0.42**	–0.12	–0.31
	Leaf thickness	**0.45**	0.01	0.23	**0.37**
	Leaf density	**0.61**	**0.79**	**0.57**	**0.61**
					
Leaf density	Leaf size	**–0.48**	**–0.46**	–0.27	**–0.38**
	Leaf thickness	–0.18	**–0.29**	**–0.46**	–0.07
					
Leaf thickness	Leaf size	**–0.32**	–0.05	0.03	0.17

* LMA  =  leaf mass per unit area.

† LDMC  =  leaf dry matter content.

Values are Pearson correlation coefficients. All trait data were corrected for variation in tree size (see Methods) and log_10_-transformed before analysis. Correlations in bold are significant at α = 0.05 after Bonferroni-Holm correction for the number of tests.

### Intraspecific correlations between leaf traits

Leaf traits were generally correlated with one another within species, and these correlations were often consistent across the four *Nothofagus* species (Table 1). Measures of leaf structural investment (LMA, LDMC, thickness and density) were usually negatively correlated with leaf size, and positively correlated with one another except for leaf density and leaf thickness, which were negatively correlated in two of the four species.

### Environmental correlates of intraspecific trait variation

**Figure 3:**
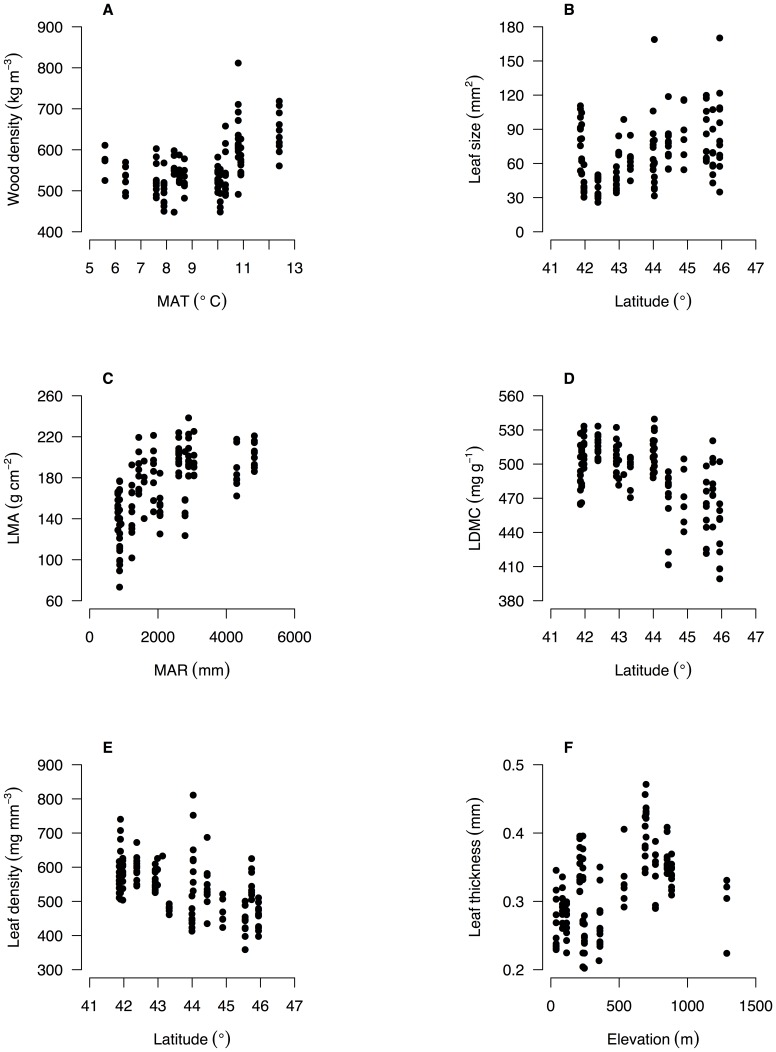
Intraspecific variation in functional traits of *Nothofagus solandri* along environmental gradients in New Zealand. Intraspecific variation in six functional traits along key environmental gradients is shown for the widespread tree species *Nothofagus solandri* throughout the South Island of New Zealand. Relationships are shown for each trait and the environmental variable with the highest Pearson correlation coefficient (Table 2). This species is illustrated as an example and all trait-environment relationships for all four species are shown in Figures S1, S2, S3, S4.

There was some evidence for consistent trait–environment relationships across species, but the most common signal from the data was for species- and trait-specific responses to environment (Tables 2 & 3; Figs. 3 & S1, S2, S3, S4). There were no trait–environment relationships that were consistent across the four species, i.e., statistically significant and in the same direction (Table 2). There were only two trait–environment relationships that shared a consistent direction across all four species and were significant for at least two of the four species. LDMC declined with soil P and this was significant for *N. solandri* and *N. fusca*, and wood density increased with MAR and this was significant for *N. menziesii* and *N. truncata* (Table 2). There was little evidence from multiple regressions that consistent combinations of environmental variables predicted intraspecific variation in a trait in all four species (Table 3). The strongest evidence came from consistently non-significant variables. For example, in three of the four species, MAR was not retained in the final multiple regression models for wood density, LMA, LDMC or leaf density. However, the variables retained in models differed among those species for those traits (Table 3).

**Table 2:** Intraspecific variation in six plant functional traits for five environmental variables in four *Nothofagus* species.

Predictor	Species	Wood density	LMA	Leaf size	LDMC	Leaf thickness	Leaf density
MAT	*N. solandri*	**0.45**	**–0.34**	0.17	–0.14	**–0.42**	0.05
	*N. menziesii*	0.23	**–0.45**	0.26	–0.23	–0.26	–0.27
	*N. fusca*	–0.12	0.19	0.06	0.15	0.34	–0.13
	*N. truncata*	–0.06	0.43	0.16	**0.50**	**0.38**	0.22
							
Elevation	*N. solandri*	**–0.37**	**0.47**	**–0.27**	**0.32**	**0.47**	0.08
	*N. menziesii*	–0.06	**0.54**	**–0.32**	**0.38**	0.23	0.41
	*N. fusca*	0.11	–0.11	0.17	0.07	**–0.39**	0.27
	*N. truncata*	–0.19	**–0.53**	0.12	**–0.54**	–0.20	**–0.51**
							
Latitude	*N. solandri*	**–0.37**	**–0.42**	**0.42**	**–0.57**	–0.06	–0.55
	*N. menziesii*	**–0.56**	0.04	0.07	–0.19	0.27	–0.21
	*N. fusca*	–0.01	–0.17	**–0.54**	**–0.39**	0.04	–0.21
	*N. truncata*	**0.42**	0.22	**–0.43**	0.14	–0.27	**0.54**
							
MAR	*N. solandri*	0.10	**0.58**	**–0.38**	**0.54**	**0.39**	0.36
	*N. menziesii*	**0.30**	0.19	–0.28	0.08	0.21	0.02
	*N. fusca*	0.25	0.20	0.21	0.27	–0.01	0.21
	*N. truncata*	**0.41**	0.17	–0.04	–0.08	–0.11	0.33
							
Soil P	*N. solandri*	–0.19	**–0.38**	**0.35**	**–0.40**	–0.08	**–0.46**
	*N. menziesii*	**–0.43**	–0.06	0.08	–0.10	–0.17	0.10
	*N. fusca*	–0.07	–0.31	–0.22	–0.49	0.12	**–0.44**
	*N. truncata*	–0.32	–0.21	0.29	–0.05	0.01	–0.29

Values are Pearson correlation coefficients. All trait data were corrected for variation in tree size (see Methods) and log_10_-transformed before analysis. Correlations in bold are significant at α = 0.05 after Bonferroni-Holm correction for the number of tests. Number of individuals per species follows Table 1.

**Table 3:** Multiple regressions predicting intraspecific variation in six plant functional traits from five environmental variables in four *Nothofagus* species.

Trait	Species	MAT	Elevation	Latitude	MAR	Soil P
Wood density	*N. solandri*	–ve	–ve	–ve	NS	NS
	*N. menziesii*	NS	NS	–ve	NS	NS
	*N. fusca*	NS	NS	NS	+ve	NS
	*N. truncata*	+ve	+ve	+ve	NS	–ve
						
Leaf size	*N. solandri*	+ve	+ve	+ve	NS	NS
	*N. menziesii*	+ve	NS	NS	–ve	NS
	*N. fusca*	NS	NS	–ve	NS	+ve
	*N. truncata*	–ve	–ve	–ve	+ve	+ve
						
LMA	*N. solandri*	–ve	NS	–ve	+ve	NS
	*N. menziesii*	NS	+ve	NS	NS	–ve
	*N. fusca*	NS	NS	NS	NS	–ve
	*N. truncata*	+ve	NS	+ve	NS	NS
						
LDMC	*N. solandri*	–ve	NS	–ve	+ve	NS
	*N. menziesii*	NS	+ve	–ve	NS	NS
	*N. fusca*	NS	NS	NS	NS	–ve
	*N. truncata*	+ve	NS	+ve	NS	NS
						
Leaf thickness	*N. solandri*	NS	+ve	NS	+ve	+ve
	*N. menziesii*	+ve	+ve	+ve	NS	–ve
	*N. fusca*	–ve	–ve	–ve	–ve	–ve
	*N. truncata*	NS	–ve	–ve	NS	NS
						
Leaf density	*N. solandri*	–ve	–ve	–ve	NS	–ve
	*N. menziesii*	–ve	NS	–ve	NS	+ve
	*N. fusca*	+ve	+ve	+ve	+ve	NS
	*N. truncata*	+ve	+ve	+ve	NS	NS

A full regression model with all five environmental variables was run for each trait and species. This model was reduced to significant terms through backwards selection. The direction of significant terms in each model is shown. Non-significant terms, removed from each model, are shown with NS. All trait data were corrected for variation in tree size (see Methods) and log_10_-transformed

**Figure 4:**
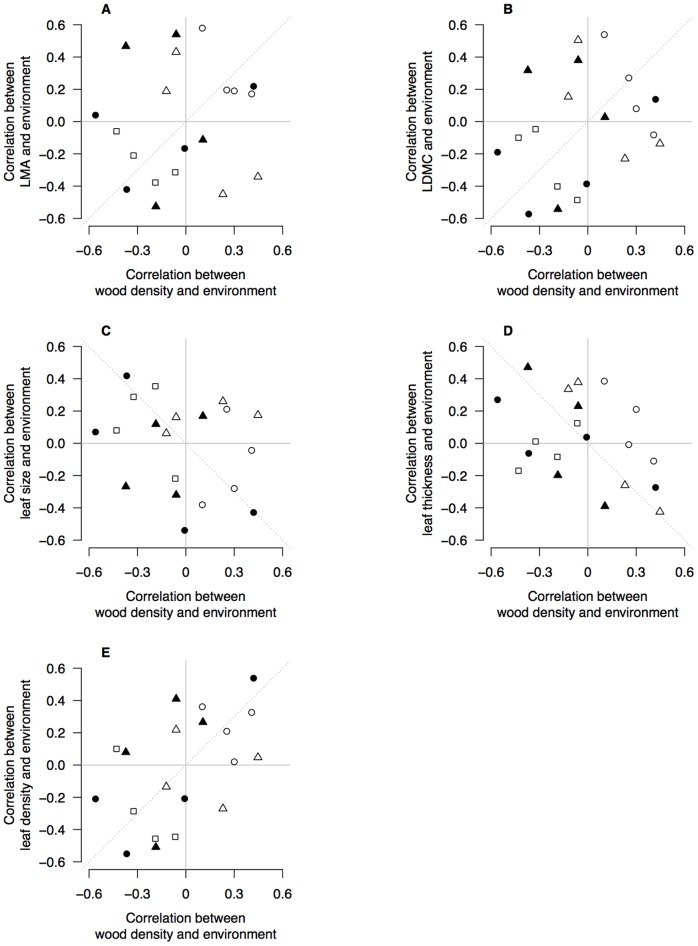
Correlation coefficients of environmental variables with wood density and leaf traits. Biplots show correlation coefficients between wood density and five environmental variables (*x*-axis), and correlation coefficients between leaf traits and environmental variables (*y*-axis). Each data point is a pair of correlation coefficients for a species. In each panel, the correlations between wood density and an environmental variable are plotted against the correlation coefficients for a leaf trait and the same environmental variable. There are four points representing each species, for each environmental variable. Open circles are correlations with MAR; filled circles are correlations with Latitude; open triangles are correlations with MAT; filled triangles are correlations with Elevation; open squares are correlations with soil P. Dashed line shows the 1∶1 relationship expected from interspecific trait correlations e.g., that wood density and LMA are positively correlated [37]–[39] and therefore so should their relationships with environment.

We plotted the correlation coefficients in Table 2 to test whether the responses within species to environment by wood density were similar to those responses by leaf traits (Fig. 4). There was no evidence that LMA, leaf size or LDMC shared responses to environment with wood density (Fig. 4A–C). However, leaf thickness and leaf density shared responses to environment with wood density (Fig. 4D–E). Leaf thickness–environment correlations were negatively correlated with wood density–environment correlations (Fig. 4D) whereas leaf density–environment correlations were positively correlated with wood density–environment correlations (Fig. 4E). This indicates that the environmental drivers of dense wood also drive thinner, denser leaves. This is confirmed by the negative relationships between leaf thickness and leaf density (Table 1).

**Figure 5:**
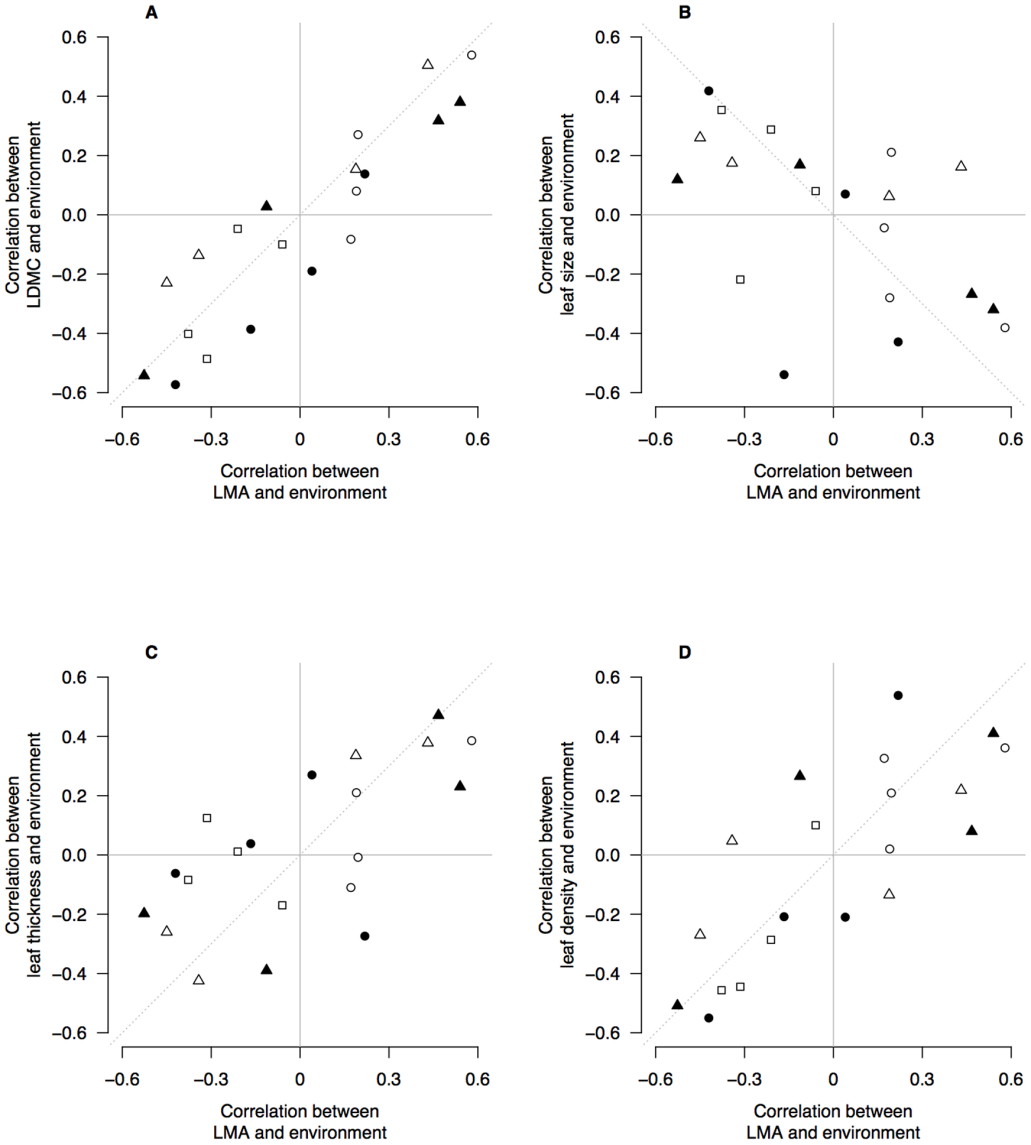
Correlation coefficients of environmental variables with leaf mass per unit area (LMA) and leaf traits. Biplots show correlation coefficients between LMA and five environmental variables (*x*-axis), and correlation coefficients between leaf traits and environmental variables (*y*-axis). Each data point is a pair of correlation coefficients for a species. In each panel, the correlations between LMA and an environmental variable are plotted against the correlation coefficients for a second leaf trait and the same environmental variable. There are four points representing each species, for each environmental variable. Open circles are correlations with MAR; filled circles are correlations with Latitude; open triangles are correlations with MAT; filled triangles are correlations with Elevation; open squares are correlations with soil P. Dashed line shows the 1∶1 relationship expected from interspecific traits correlations, e.g., that LMA and leaf size are negatively correlated [31] and therefore so should their relationships with environment.

We used a similar approach to evaluate whether LMA–environment correlations were shared with the component leaf traits that underpin LMA (Fig. 5). There was very strong evidence that LDMC–environment correlations and leaf density–environment correlations were both similar to LMA–environment correlations (Fig. 5A, D). This was also true for leaf thickness (Fig. 5C) but the association was weaker. Leaf size – environment correlations were weakly and negatively associated with LMA–environment correlations (Fig. 5B). The environmental conditions promoting higher LMA within species also tend towards smaller leaves, but the relationship is weak.

## Discussion

### Intraspecific correlations between wood density and leaf traits, and among leaf traits

Interspecific studies suggest that trait variation is coordinated among tissues, leading to the emergence of trait syndromes and economic spectra that reflect whole-plant strategies. Our data clearly demonstrate that intraspecific leaf-trait variation is strongly correlated among traits, confirming that the leaf economics spectrum applies within species. However, we found limited evidence for close coordination between leaf traits and wood density. In contrast to most interspecific trait studies [31], [34], [39], we found no evidence that leaf size and LMA were correlated with wood density for *Nothofagus* species. However, we found weak evidence that both LDMC and leaf density were positively correlated with wood density within species, a result concordant with interspecific studies [37], [42] that points to shared responses of leaves and wood for dry matter allocation relative to leaf or stem volume. These findings support the hypothesis that LMA is not a surrogate for measuring the component traits underpinning it. Two of the component traits (LDMC and leaf density) correlated with wood density while the integrated trait (LMA) did not. This highlights the need to measure the components of LMA in order to determine how leaf construction covaries with other plant traits [26]. Trait-specific coupling of leaf and wood traits within species questions the general applicability of a whole-plant trait economics spectrum [3] and supports the view that leaf and stem trait variation is largely uncoupled [34].

### Why aren't leaf traits and wood density correlated within species?

Traits may be uncoordinated within species because the constraints driving each trait operate over different parts of the same environmental gradient [16]. Alternatively it may be because of contrasting, non-linear responses by traits to environment [53]. For example, the relationship between wood density and leaf size might represent a strategy to avoid water loss, but leaf size may only decline with increasing wood density in large-leaved species exposed to seasonal water stress. The absence of a relationship between wood density and leaf size in any of the four evergreen *Nothofagus* species assessed here may reflect low moisture stress in New Zealand's temperate rainforests or the limited influence of moisture stress on leaf size in evergreen species having relatively small (<700 mm^2^) leaves (Table S3). Lastly, variation in wood density will reflect interannual variation in tree growth rate throughout the sampled core spanning many years, while traits of current-year leaves will be responding more directly to the environment in that year, to optimise resource acquisition and expenditure.

### Sources of intraspecific variation

An unresolved question is whether intraspecific variation reflects genetic polymorphism, phenotypic plasticity, or a combination of the two. Widespread species might be inherently more variable because they are more numerous, and therefore support greater genetic polymorphism, or alternatively, because they encounter a wider diversity of environments. Common garden experiments can be used to minimise the effects of phenotypic plasticity and isolate those of genetic polymorphism. A provenance trial to examine genetic polymorphism in New Zealand *Nothofagus* species sampled elevational and latitudinal gradients and demonstrated substantial genetic polymorphism in seedling growth rate, leaf size and leaf shape in the two most widespread species (*N. solandri* and *N. menziesii*), and moderate polymorphism in the less widespread *N. fusca* [54]. In order to generalise beyond empirical studies, we need to establish whether large intraspecific variation is driven by environmental heterogeneity or simply by population size and demographic history [8]. Both of these factors should increase with geographic range size, and are not easily partitioned out. However, a controlled experiment to compare intraspecific trait variation of Australian tree species with contrasting range sizes found no evidence that widespread species had higher intraspecific variation [55]. Although it is clear that there is substantial intraspecific variation in plant functional traits among sites that differs among species, further work is needed to better understand the sources and drivers of this variation, specifically the contributions by environment and genetic variation to trait variation.

### Intraspecific trait–environment relationships

Variation in a trait was not usually driven by the same environmental variable across all four *Nothofagus* species. There were only two trait–environment relationships that shared a consistent direction within each of the four species, while being statistically significant in at least two species. Results from individual species were often concordant with models of trait variation developed using interspecific relationships between the six traits and five environmental variables examined here. Interspecific studies of wood density variation and environment suggest that wood density should be greatest at high temperatures (i.e., low latitude and low elevation) [29], [56], [57], on relatively dry sites [58]–[60], and on infertile soils [61]–[63]. Wood density of two species (*N. menziesii* and *N. solandri*) increased with temperature (or more accurately, decreasing latitude and increasing MAT). However, wood density of *N. truncata* increased with latitude, contrary to expectation. Wood density of two species increased with mean annual rainfall (MAR), counter to predictions from interspecific studies. Rainfall gradients in southern New Zealand range from 668 to 4875 mm yr^–1^ (Table S1) with only six sites receiving less than 1000 mm yr^−1^. We propose that the relationship between moisture availability and wood density is strongly non-linear with both moisture-limited and excessively wet sites favouring dense wood. High rainfall is associated with two factors that limit tree growth rates and hence increase wood density. The first is nutrient leaching and reduced soil fertility. For example, in our study MAR and soil total P were negatively correlated (Table S2), providing strong support for this mechanism. The second is low solar radiation that would limit canopy photosynthesis and hence tree growth. Relatively few studies explicitly model the effect of measured soil fertility yet we demonstrate how this can determine variation in leaf structural traits such as LMA and LDMC [64], as well as wood density. Across all four species, models using indirect measures of environmental variation (elevation and latitude) were commonly stronger predictors of trait variation than direct measures (MAT and MAR, and also solar radiation (not shown)), suggesting that indirect gradients capture aspects of growing season length and photosynthetic potential that are crucial for determining trait variation in addition to the direct effects of temperature and rainfall.

Leaf thickness declined with MAT in the two small-leaved species (*N. menziesii* and *N. solandri*) but increased with MAT in the two large-leaved species (*N. fusca* and *N. truncata*), suggesting that allocation to leaf thickness within a species is contingent on leaf size and the predominant environmental niche of a species (cool for the first pair, and warmer for the second). Intriguingly, there is no consensus on how leaf thickness varies among species at a global scale with temperature – both positive [65] and negative [66] relationships have been reported. Thick leaves clearly arise across a range of climatic situations through allocation to photosynthetically-active mesophyll cells [65], protective epidermal layers [67], or both, and these allocation patterns are not in-common across species.

Our data provide some support to the hypothesis that suites of traits are correlated within species because of shared responses to strong environmental gradients [33], [68] but see [69]. There were several instances where four of the six traits were significantly correlated with a single environmental factor within a species (e.g., elevation in *N. solandri* and *N. menziesii*). This was particularly strong for leaf traits (Fig. 5) where the environmental drivers of variation were shared between LMA and LDMC, leaf density and leaf thickness. By and large, however, the environmental drivers of variation in wood density were not shared with leaf traits (Fig. 4). The exception to this was the link between wood density, and leaf density and leaf thickness. Environments generating dense wood also selected for denser, thinner leaves, but not higher LMA, LDMC or smaller leaves. The strongest environmental drivers of high wood density, broadly speaking, were cooler and wetter climates and less fertile soils. Such conditions are known to promote thinner leaves, particularly when associated with low solar radiation [25], [65] and low soil fertility, which reduces the fraction of intercellular space in leaves and hence increases leaf density [70].

Intraspecific covariation in wood density and leaf traits among sites and with environment did not mirror results reported among species from large-scale studies encompassing larger global environmental gradients [2], [56]. Large-scale studies tend to capture the ‘end-points’ of trait variation; e.g., at large latitudinal scales, large leaves and dense wood from the wet tropics are compared with needle leaves and low density softwoods from boreal conifer forests. Trait covariation with environment is an almost inevitable consequence at such large spatial scales. At smaller spatial scales, such as those in this study, trait covariation with environment may not be observed if one or either trait only responds over a portion of the environmental gradient. Optimal combinations of traits may vary along complex environmental gradients and thus traits may be somewhat decoupled within a species throughout their range. The probability of detecting relationships between multiple traits and environment will vary according to where species occur and are sampled [68]. In this study, some traits varied little within species (Table S3), while for others, the amount of intraspecific trait variation was comparable with interspecific variation at similar spatial scales, in similar environments. For example, wood density of *N. solandri* varied 1.8-fold from 448 kg m^−3^ to 811 kg m^−3^ (Table S3) spanning much of the range of this trait across New Zealand forests (2.9–fold from 326 kg m^−3^ to 930 kg m^−3^, *N* = 130 species, unpublished data of the authors), and indeed globally [30]. These observations point to different mechanisms driving trait variation: for global comparisons, this is species turnover of plants having markedly different strategies or trait syndromes whereas for intraspecific variation, the mechanism is the coordinated response within a species along environmental gradients throughout its range. Our findings contribute to the growing understanding of how different mechanisms drive variation in plant functional traits at local (within community), regional (among communities within a biome) and global scales. Lastly, our study was confined to four closely-related tree species over spatial scales of <1000 km. Our data may not be representative of the amount of intraspecific variation typical of all species, across different plant growth forms, and at contrasting locations along global environmental gradients. Further studies of widely distributed species, particularly those spanning biomes will provide a valuable contrast with our data [19].

## Supporting Information

Figures S1 **Intraspecific variation in six functional traits along five environmental gradients for** ***Nothofagus solandri*** **.**
(TIFF)

Figures S2 **Intraspecific variation in six functional traits along five environmental gradients for** ***Nothofagus menziesii*** **.**
(TIFF)

Figures S3 **Intraspecific variation in six functional traits along five environmental gradients** ***Nothofagus fusca*** **.**
(TIFF)

Figures S4 **Intraspecific variation in six functional traits along five environmental gradients for** ***Nothofagus truncata*** **.**
(TIFF)

Table S1 **Thirty sites in southern New Zealand sampled for** ***Nothofagus*** **wood density and leaf structural traits.**
(DOCX)

Table S2 **Pearson's correlation coefficients between environmental variables used to predict trait variation.**
(DOCX)

Table S3 **Inter/intraspecific variation in** ***Nothofagus*** **wood density and leaf traits sampled throughout southern New Zealand.**
(DOCX)

Table S4 **Wood density and leaf trait data sampled in** ***Nothofagus*** **throughout southern New Zealand.**
(DOCX)
