# Supplementary figures and images for: Intraspecific Relationships among Wood Density, Leaf Structural Traits and Environment in Four Co-Occurring Species of Nothofagus in New Zealand
Source: PLoS One. 2013 Mar 18;8(3):e58878. doi: 10.1371/journal.pone.0058878 (PMC3601108; doi:10.1371/journal.pone.0058878)

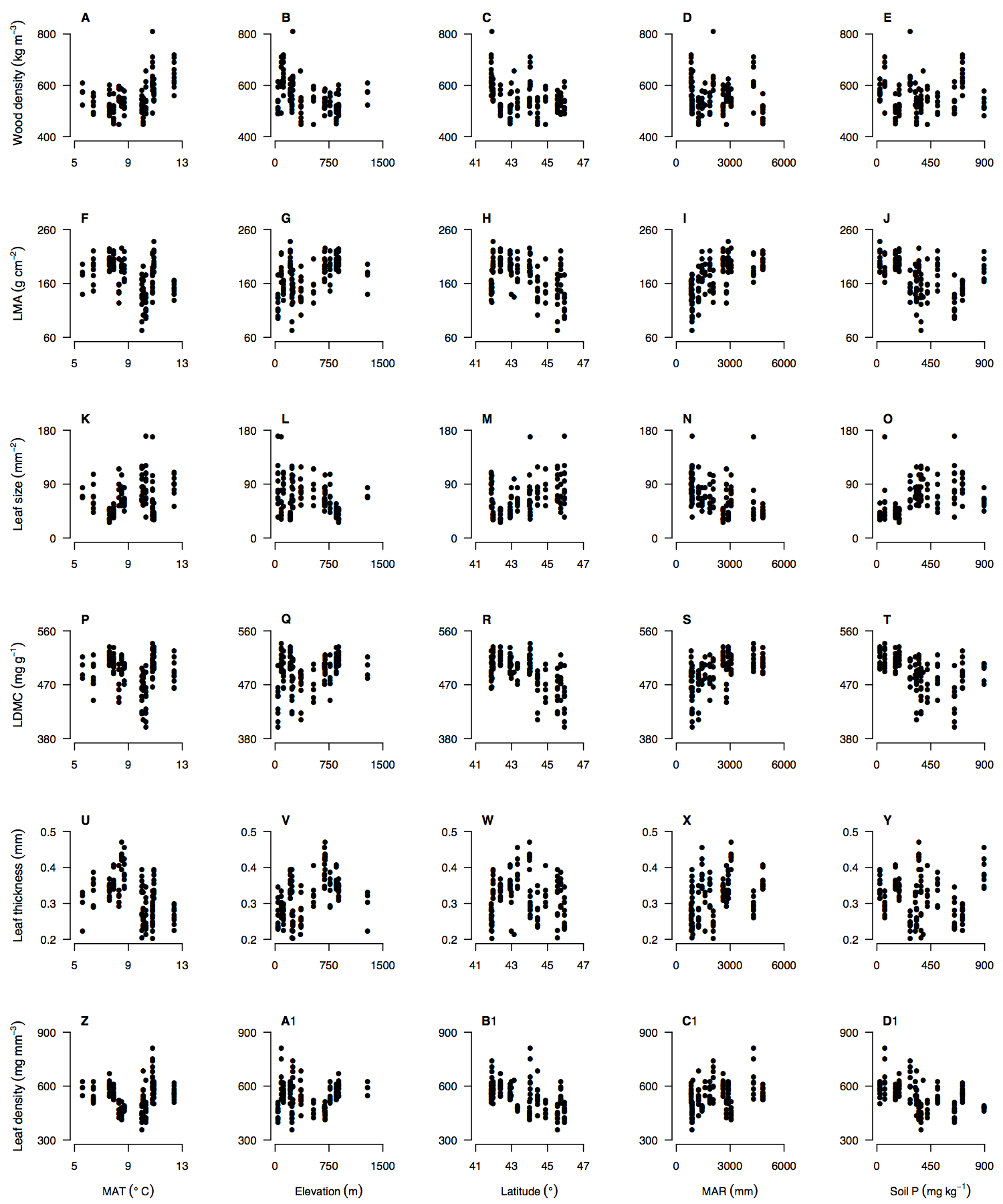

Supplement: Figures S1 — Intraspecific variation in six functional traits along five environmental gradients for Nothofagus solandri . (TIFF) [file pone.0058878.s001.tif]

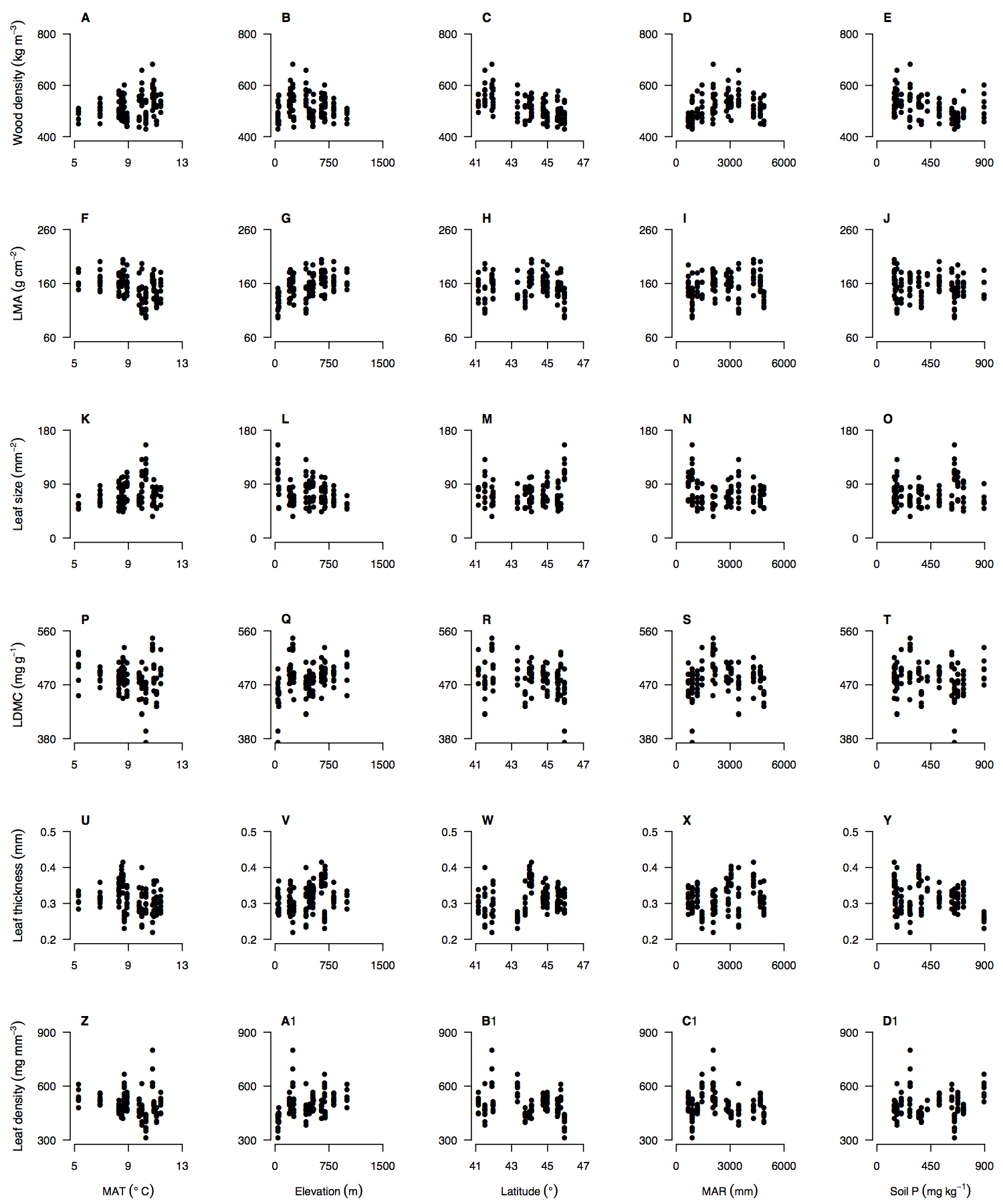

Supplement: Figures S2 — Intraspecific variation in six functional traits along five environmental gradients for Nothofagus menziesii . (TIFF) [file pone.0058878.s002.tif]

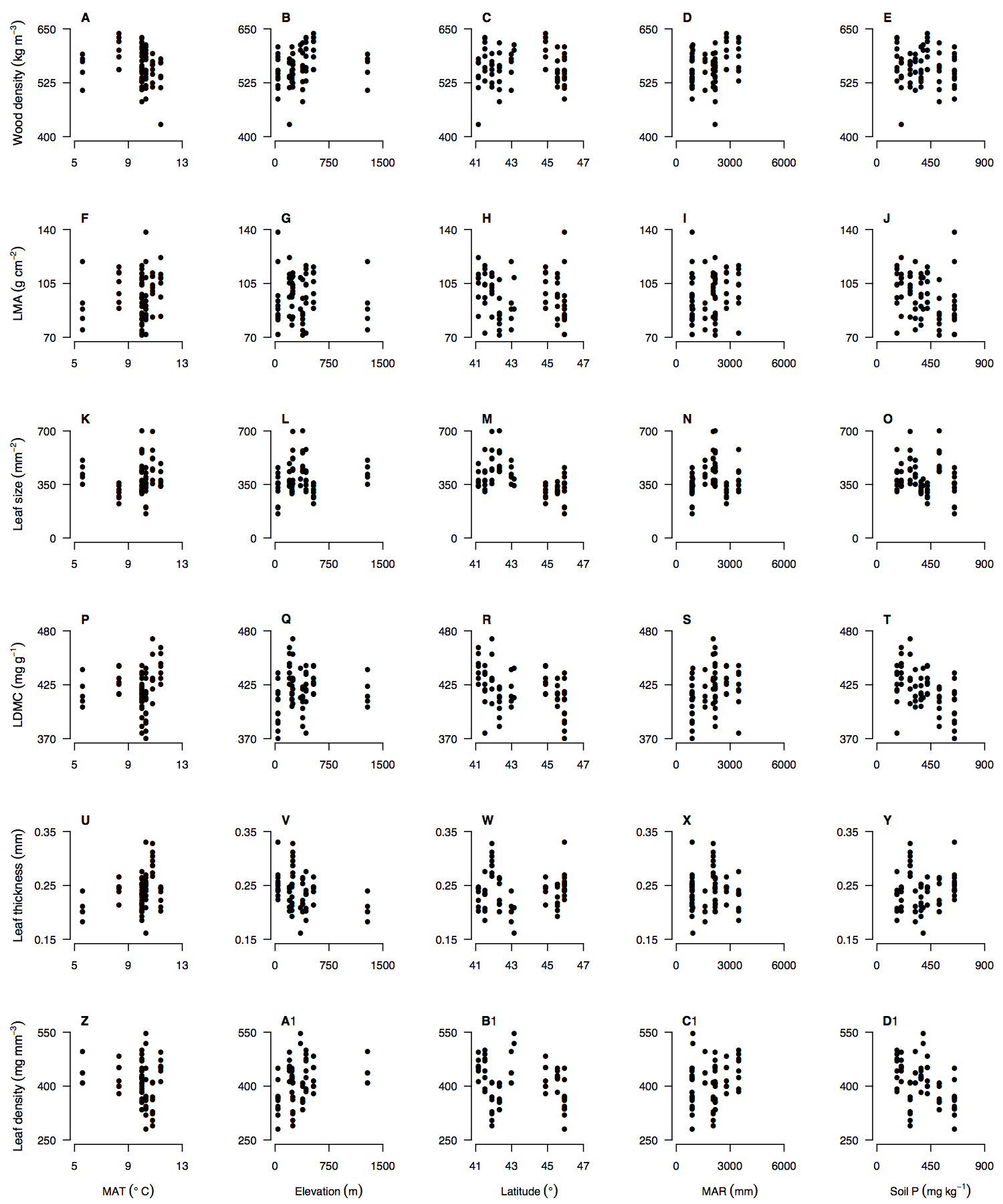

Supplement: Figures S3 — Intraspecific variation in six functional traits along five environmental gradients Nothofagus fusca . (TIFF) [file pone.0058878.s003.tif]

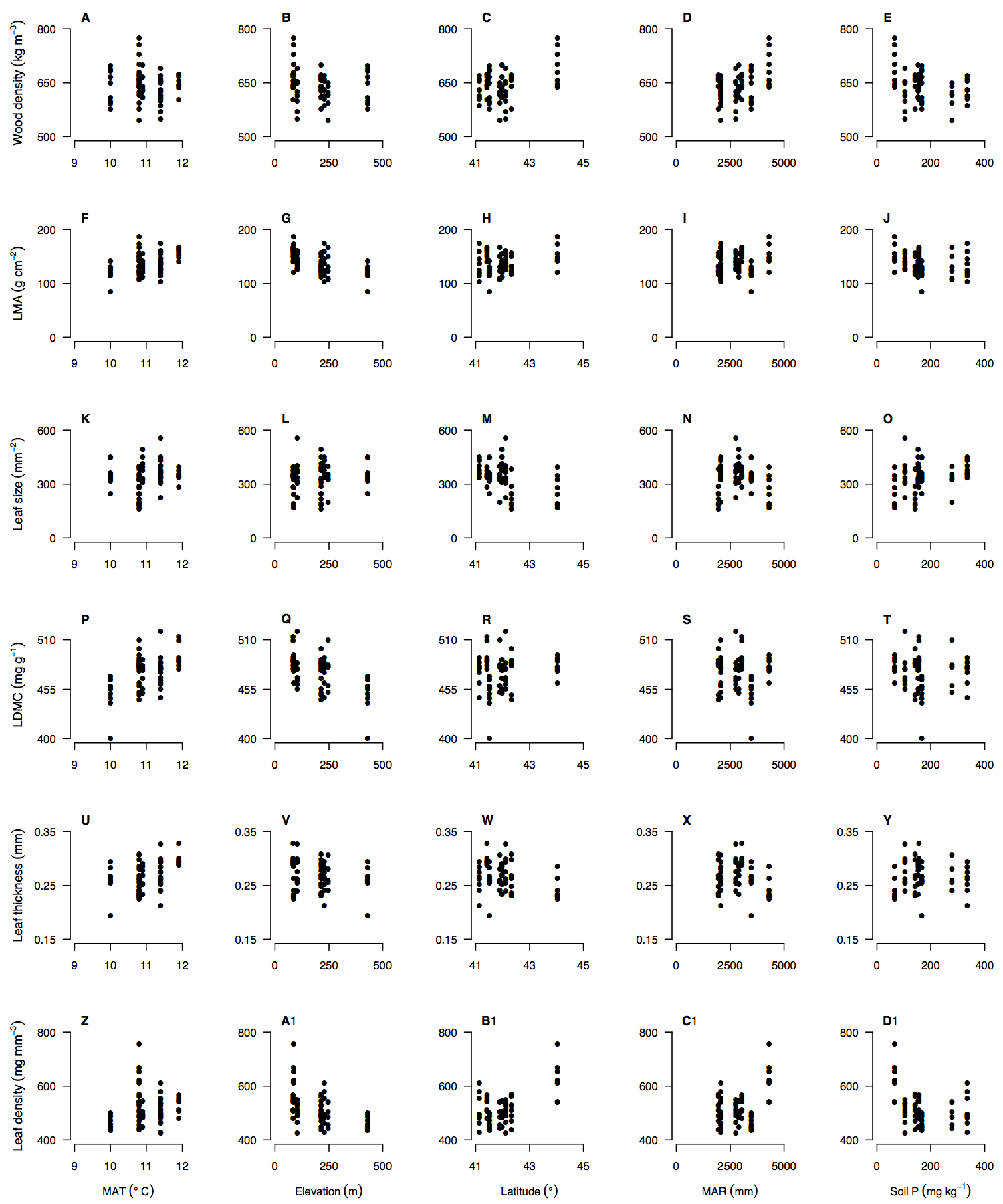

Supplement: Figures S4 — Intraspecific variation in six functional traits along five environmental gradients for Nothofagus truncata . (TIFF) [file pone.0058878.s004.tif]
