# Supplementary material for: Intraspecific Relationships among Wood Density, Leaf Structural Traits and Environment in Four Co-Occurring Species of Nothofagus in New Zealand
Source: PLoS One. 2013 Mar 18;8(3):e58878. doi: 10.1371/journal.pone.0058878 (PMC3601108; doi:10.1371/journal.pone.0058878)
Supplement: Table S1 — Thirty sites in southern New Zealand sampled for Nothofagus wood density and leaf structural traits. (DOCX) [file pone.0058878.s005.docx]

**Table S1.** **Thirty sites in southern New Zealand sampled for *Nothofagus* wood density and leaf structural traits.**

| **Site** | **MAT**  **(°C)** | **MAR**  **(mm)** | **Latitude** | **Elevation** | **Soil P**  **(mg kg^–1^)** | ***N. menziesii*** | ***N. solandri*** | ***N. fusca*** | ***N. truncata*** |
| --- | --- | --- | --- | --- | --- | --- | --- | --- | --- |
| Mt Burns | 5.3 | 2034 | −45.7 | 1001 | 625 | • |  |  |  |
| Woolshed Hill | 5.6 | 1597 | −43.0 | 1288 | 322 |  | • | • |  |
| Green Lake | 6.4 | 1869 | −45.7 | 766 | 507 |  | • |  |  |
| Gertrude Valley | 6.9 | 4680 | −44.8 | 817 | 521 | • |  |  |  |
| Lewis Pass | 7.6 | 2608 | −42.4 | 880 | 185 |  | • |  |  |
| Arthur’s Pass | 7.9 | 4821 | −42.9 | 851 | 155 |  | • |  |  |
| Princhester Hut | 8.3 | 1170 | −45.6 | 477 | 723 | • |  |  |  |
| Eglinton Valley | 8.3 | 2792 | −44.9 | 536 | 421 | • | • | • |  |
| Huxley River | 8.5 | 3055 | −44.0 | 697 | 350 | • | • |  |  |
| Haast Pass | 8.6 | 4303 | −44.1 | 647 | 146 | • |  |  |  |
| Lake Heron Station | 8.7 | 1438 | −43.3 | 690 | 896 | • | • |  |  |
| Roaring Meg | 8.9 | 668 | −45.0 | 525 | 676 | • |  |  |  |
| Waikaia slopes | 9.8 | 892 | −45.6 | 261 | 274 | • |  |  |  |
| Piano Flat | 10 | 878 | −45.6 | 235 | 369 |  | • | • |  |
| Staircase Creek | 10.0 | 2168 | −42.3 | 385 | 521 |  |  | • |  |
| Karamea Bluff | 10.0 | 3477 | −41.5 | 445 | 167 | • |  | • | • |
| Kidds Bush | 10.1 | 1238 | −44.4 | 360 | 331 |  | • |  |  |
| Tom Creek | 10.3 | 883 | −46.0 | 39 | 648 | • | • | • |  |
| Mt Grey | 10.3 | 920 | −43.1 | 353 | 387 |  | • | • |  |
| Granville | 10.8 | 1964 | −42.3 | 200 | 142 |  |  |  | • |
| Peasoup Creek | 10.8 | 2060 | −41.9 | 240 | 278 | • | • | • | • |
| Macfarlane Mound | 10.8 | 4304 | −44.0 | 85 | 66 |  | • |  | • |
| Charleston forest | 10.9 | 2891 | −42.0 | 220 | 153 | • |  |  | • |
| Charleston woodland | 10.9 | 2899 | −42.0 | 200 | 25 |  | • |  |  |
| Paringa | 11.1 | 4875 | −43.8 | 51 | 371 | • |  |  |  |
| Ronga Saddle | 11.4 | 2073 | −41.1 | 245 | 335 |  |  |  | • |
| Oparara | 11.4 | 2161 | −41.2 | 200 | 203 | • |  | • |  |
| Bullock Creek | 11.4 | 2756 | −42.1 | 105 | 105 |  |  |  | • |
| Lake Hanlon | 11.9 | 3034 | −41.4 | 100 | 156 |  |  |  | • |
| Mirza Creek | 12.4 | 825 | −41.9 | 120 | 716 |  | • |  |  |

Individual trees (*n* = 9–11 were sampled within each site for each *Nothofagus* species present. MAT and MAR were modelled using thin-plate splines (Leathwick JR, Overton JM, McLeod M. 2003. An environmental domain classification of New Zealand and its use as a tool for biodiversity management. Conserv Biol 17: 1612-1623).
