# Supplementary material for: Intraspecific Relationships among Wood Density, Leaf Structural Traits and Environment in Four Co-Occurring Species of Nothofagus in New Zealand
Source: PLoS One. 2013 Mar 18;8(3):e58878. doi: 10.1371/journal.pone.0058878 (PMC3601108; doi:10.1371/journal.pone.0058878)
Supplement: Table S2 — Pearson's correlation coefficients between environmental variables used to predict trait variation. (DOCX) [file pone.0058878.s006.docx]

**Table S2. Pearson’s correlation coefficients between environmental variables used to predict trait variation.**

|  | **Latitude (°)** | **Soil total P (mg.kg^–1^)** | **MAT (°C)** | **MAR (mm)** |
| --- | --- | --- | --- | --- |
| Soil total P (mg kg^–1^) | **0.48** |  |  |  |
| MAT (°C) | **−0.54** | −0.31 |  |  |
| MAR (mm) | −0.21 | **−0.50** | −0.06 |  |
| Elevation (m) | 0.26 | 0.22 | **−0.93** | 0.09 |

MAT = mean annual temperature; MAR = mean annual rainfall.

*N* = 30. Coefficients in bold are significant at *P*< 0.05.
