# Supplementary material for: Intraspecific Relationships among Wood Density, Leaf Structural Traits and Environment in Four Co-Occurring Species of Nothofagus in New Zealand
Source: PLoS One. 2013 Mar 18;8(3):e58878. doi: 10.1371/journal.pone.0058878 (PMC3601108; doi:10.1371/journal.pone.0058878)
Supplement: Table S3 — Inter/intraspecific variation in Nothofagus wood density and leaf traits sampled throughout southern New Zealand. (DOCX) [file pone.0058878.s007.docx]

**Table S3.** **Inter/intraspecific variation in *Nothofagus* wood density and leaf traits sampled throughout southern New Zealand.**

| **Trait** | **Species** | ***N**** | **Mean (± 1SD)** | **CV%** | **Range of values** | **Max – Min** | **Max/Min** |
| --- | --- | --- | --- | --- | --- | --- | --- |
| Wood density | *N. menziesii* | 119 | 516 (48) | 9.2 | 430–683 | 253 | 1.6 |
| (kg m^–3^) | *N. solandri* | 128 | 556 (62) | 11.1 | 448–811 | 363 | 1.8 |
|  | *N. fusca* | 65 | 562 (41) | 7.2 | 429–640 | 211 | 1.5 |
|  | *N. truncata* | 67 | 643 (43) | 6.7 | 546–776 | 230 | 1.4 |
| Leaf size | *N. menziesii* | 119 | 76 (21) | 27.8 | 36–156 | 120 | 4.3 |
| (mm^2^) | *N. solandri* | 128 | 68 (28) | 40.8 | 26–170 | 144 | 6.6 |
|  | *N. fusca* | 65 | 390 (106) | 27.1 | 159–704 | 545 | 4.4 |
|  | *N. truncata* | 67 | 337 (81) | 24.2 | 164–559 | 395 | 3.4 |
| LMA‡ | *N. menziesii* | 119 | 154 (24) | 15.3 | 96–204 | 108 | 2.1 |
| (g cm^–2^) | *N. solandri* | 128 | 171 (34) | 19.9 | 73–239 | 166 | 3.3 |
|  | *N. fusca* | 65 | 97 (15) | 15.1 | 71–139 | 68 | 1.9 |
|  | *N. truncata* | 67 | 138 (19) | 14.1 | 85–187 | 102 | 2.2 |
| LDMC¶ | *N. menziesii* | 119 | 481 (28) | 5.7 | 374–549 | 175 | 1.5 |
| (mg g^–1^) | *N. solandri* | 128 | 490 (29) | 6.0 | 399–540 | 141 | 1.4 |
|  | *N. fusca* | 65 | 420 (21) | 5.0 | 370–472 | 102 | 1.3 |
|  | *N. truncata* | 67 | 475 (19) | 4.1 | 401–520 | 119 | 1.3 |
| Leaf density | *N. menziesii* | 119 | 493 (70) | 14.2 | 314–801 | 487 | 2.5 |
| (mg g^–3^) | *N. solandri* | 128 | 540 (79) | 14.7 | 359–811 | 452 | 2.3 |
|  | *N. fusca* | 65 | 408 (57) | 14.1 | 280–546 | 266 | 2.0 |
|  | *N. truncata* | 67 | 517 (62) | 11.9 | 426–755 | 329 | 1.8 |
| Leaf thickness | *N. menziesii* | 119 | 0.31 (0.04) | 12.7 | 0.22–0.41 | 0.19 | 1.9 |
| (mm) | *N. solandri* | 128 | 0.32 (0.06) | 19.0 | 0.20–0.47 | 0.27 | 2.3 |
|  | *N. fusca* | 65 | 0.24 (0.03) | 14.1 | 0.16–0.33 | 0.17 | 2.0 |
|  | *N. truncata* | 67 | 0.27 (0.03) | 10.3 | 0.19–0.33 | 0.14 | 1.7 |

** N* = number of individual trees sampled.

‡ LMA = leaf mass per unit area.

¶ LDMC = leaf dry matter content.
