# Supplementary material for: Intraspecific Relationships among Wood Density, Leaf Structural Traits and Environment in Four Co-Occurring Species of Nothofagus in New Zealand
Source: PLoS One. 2013 Mar 18;8(3):e58878. doi: 10.1371/journal.pone.0058878 (PMC3601108; doi:10.1371/journal.pone.0058878)
Supplement: Table S4 — Wood density and leaf trait data sampled in Nothofagus throughout southern New Zealand. (DOCX) [file pone.0058878.s008.docx]

**Table S4.** **Wood density and leaf trait data sampled in *Nothofagus* throughout southern New Zealand.**

| **Site** | **Species** | **DBH**  **(mm)** | **Leaf size**  **(mm^2^)** | **LDMC**  **(mg g^-1^)** | **LMA**  **(g cm^-2^)** | **Leaf**  **thickness**  **(mm)** | **Leaf**  **density**  **(mg mm^-3^)** | **Wood**  **density**  **(kg m^-3^)** |
| --- | --- | --- | --- | --- | --- | --- | --- | --- |
| Arthur’s Pass | *N. solandri* | 239 | 52.4 | 505.5 | 221.0 | 0.409 | 540.8 | 519.0 |
| Arthur’s Pass | *N. solandri* | 97 | 46.5 | 489.4 | 190.1 | 0.349 | 545.0 | 495.1 |
| Arthur’s Pass | *N. solandri* | 117 | 41.7 | 512.4 | 206.0 | 0.348 | 592.3 | 505.0 |
| Arthur’s Pass | *N. solandri* | 96 | 38.1 | 500.0 | 186.2 | 0.353 | 526.8 | 514.0 |
| Arthur’s Pass | *N. solandri* | 126 | 47.6 | 502.5 | 214.3 | 0.402 | 532.8 | 517.7 |
| Arthur’s Pass | *N. solandri* | 183 | 41.0 | 508.2 | 216.0 | 0.354 | 609.6 | 471.0 |
| Arthur’s Pass | *N. solandri* | 84 | 45.5 | 522.1 | 199.7 | 0.341 | 585.9 | 450.2 |
| Arthur’s Pass | *N. solandri* | 189 | 34.2 | 532.3 | 193.0 | 0.352 | 548.2 | 520.4 |
| Arthur’s Pass | *N. solandri* | 90 | 36.8 | 515.3 | 190.0 | 0.341 | 557.6 | 568.0 |
| Arthur’s Pass | *N. solandri* | 87 | 36.0 | 500.0 | 204.5 | 0.361 | 566.0 | 462.4 |
| Arthur’s Pass | *N. solandri* | 75 | 57.3 | 492.5 | 192.1 | 0.365 | 525.8 | 472.5 |
| Charleston woodland | *N. solandri* | 134 | 35.7 | 500.0 | 182.1 | 0.314 | 579.5 | 605.0 |
| Charleston woodland | *N. solandri* | 201 | 59.0 | 529.7 | 196.6 | 0.336 | 585.5 | 626.4 |
| Charleston woodland | *N. solandri* | 135 | 43.6 | 507.3 | 238.5 | 0.395 | 603.3 | 569.6 |
| Charleston woodland | *N. solandri* | 238 | 34.5 | 533.3 | 208.7 | 0.337 | 620.0 | 541.4 |
| Charleston woodland | *N. solandri* | 166 | 30.6 | 504.3 | 192.8 | 0.328 | 587.1 | 586.7 |
| Charleston woodland | *N. solandri* | 155 | 30.3 | 516.9 | 201.3 | 0.331 | 607.5 | 538.5 |
| Charleston woodland | *N. solandri* | 137 | 34.7 | 496.4 | 196.0 | 0.365 | 537.5 | 575.4 |
| Charleston woodland | *N. solandri* | 118 | 39.3 | 524.4 | 218.8 | 0.392 | 558.5 | 545.3 |
| Charleston woodland | *N. solandri* | 90 | 39.8 | 500.0 | 191.0 | 0.379 | 503.8 | 570.0 |
| Charleston woodland | *N. solandri* | 65 | 39.1 | 496.5 | 181.6 | 0.315 | 576.5 | 544.9 |
| Charleston woodland | *N. solandri* | 74 | 38.2 | 518.3 | 222.5 | 0.355 | 626.1 | 563.0 |
| Eglington Valley | *N. solandri* | 416 | 67.7 | 471.4 | 158.1 | 0.337 | 469.3 | 586.3 |
| Eglington Valley | *N. solandri* | 294 | 54.5 | 504.5 | 205.5 | 0.406 | 506.7 | 597.8 |
| Eglington Valley | *N. solandri* | 465 | 89.5 | 462.5 | 158.7 | 0.304 | 521.2 | 549.4 |
| Eglington Valley | *N. solandri* | 336 | 116.1 | 495.5 | 143.0 | 0.320 | 447.4 | 540.2 |
| Eglington Valley | *N. solandri* | 544 | 115.4 | 449.2 | 145.6 | 0.325 | 448.5 | 555.2 |
| Eglington Valley | *N. solandri* | 61 | 80.9 | 440.5 | 123.6 | 0.292 | 423.6 | 447.9 |
| Green Lake | *N. solandri* | 654 | 42.9 | 520.5 | 221.4 | 0.354 | 625.3 | 521.6 |
| Green Lake | *N. solandri* | 320 | 58.6 | 472.8 | 175.2 | 0.294 | 595.5 | 558.5 |
| Green Lake | *N. solandri* | 347 | 90.2 | 482.8 | 186.3 | 0.358 | 519.7 | 487.1 |
| Green Lake | *N. solandri* | 173 | 69.7 | 501.5 | 193.8 | 0.368 | 526.3 | 558.8 |
| Green Lake | *N. solandri* | 215 | 57.6 | 505.1 | 157.7 | 0.290 | 544.3 | 495.8 |
| Green Lake | *N. solandri* | 154 | 69.1 | 472.4 | 206.3 | 0.388 | 531.9 | 536.8 |
| Green Lake | *N. solandri* | 263 | 107.3 | 444.7 | 146.7 | 0.290 | 505.3 | 538.2 |
| Green Lake | *N. solandri* | 303 | 50.2 | 478.3 | 197.2 | 0.337 | 585.2 | 569.9 |
| Lake Heron Station | *N. solandri* | 615 | 57.9 | 506.0 | 219.3 | 0.456 | 480.6 | 578.0 |
| Lake Heron Station | *N. solandri* | 469 | 61.5 | 497.6 | 167.5 | 0.348 | 481.8 | 512.0 |
| Lake Heron Station | *N. solandri* | 196 | 54.8 | 476.9 | 188.0 | 0.381 | 493.1 | 550.0 |
| Lake Heron Station | *N. solandri* | 185 | 84.7 | 501.8 | 168.8 | 0.366 | 460.8 | 482.2 |
| Lake Heron Station | *N. solandri* | 352 | 65.0 | 506.0 | 193.8 | 0.410 | 472.8 | 513.8 |
| Lake Heron Station | *N. solandri* | 301 | 64.6 | 504.8 | 164.1 | 0.342 | 479.8 | 513.2 |
| Lake Heron Station | *N. solandri* | 528 | 44.8 | 500.0 | 205.4 | 0.424 | 484.3 | 518.7 |
| Lake Heron Station | *N. solandri* | 385 | 66.1 | 470.6 | 181.5 | 0.378 | 480.0 | 535.3 |
| Huxley River | *N. solandri* | 151 | 106.1 | 487.9 | 190.6 | 0.437 | 436.3 | 549.3 |
| Huxley River | *N. solandri* | 156 | 74.1 | 505.4 | 190.3 | 0.428 | 444.6 | 587.3 |
| Huxley River | *N. solandri* | 164 | 79.7 | 506.3 | 202.0 | 0.422 | 479.1 | 543.4 |
| Huxley River | *N. solandri* | 317 | 63.5 | 495.7 | 182.7 | 0.394 | 463.4 | 551.2 |
| Huxley River | *N. solandri* | 243 | 58.2 | 520.7 | 194.2 | 0.432 | 449.4 | 519.9 |
| Huxley River | *N. solandri* | 332 | 76.5 | 491.2 | 181.9 | 0.430 | 423.1 | 526.0 |
| Huxley River | *N. solandri* | 372 | 54.5 | 512.1 | 194.5 | 0.471 | 412.8 | 536.7 |
| Huxley River | *N. solandri* | 591 | 86.1 | 517.3 | 225.3 | 0.437 | 515.8 | 521.3 |
| Kidds Bush | *N. solandri* | 349 | 79.3 | 471.2 | 165.2 | 0.287 | 576.4 | 542.4 |
| Kidds Bush | *N. solandri* | 157 | 66.5 | 474.2 | 151.9 | 0.284 | 535.2 | 540.7 |
| Kidds Bush | *N. solandri* | 499 | 84.5 | 483.4 | 172.8 | 0.331 | 521.7 | 552.6 |
| Kidds Bush | *N. solandri* | 114 | 55.3 | 487.3 | 133.5 | 0.257 | 519.5 | 518.6 |
| Kidds Bush | *N. solandri* | 301 | 68.2 | 481.1 | 130.5 | 0.238 | 549.2 | 459.6 |
| Kidds Bush | *N. solandri* | 462 | 84.2 | 488.2 | 192.5 | 0.350 | 549.8 | 473.5 |
| Kidds Bush | *N. solandri* | 352 | 118.8 | 411.6 | 101.9 | 0.234 | 434.9 | 514.9 |
| Kidds Bush | *N. solandri* | 339 | 75.1 | 493.4 | 165.7 | 0.241 | 687.5 | 538.8 |
| Kidds Bush | *N. solandri* | 107 | 77.9 | 461.1 | 146.8 | 0.252 | 581.6 | 448.4 |
| Kidds Bush | *N. solandri* | 74 | 86.1 | 481.9 | 126.7 | 0.240 | 527.5 | 493.8 |
| Kidds Bush | *N. solandri* | 76 | 54.9 | 422.7 | 130.3 | 0.261 | 499.4 | 521.6 |
| Lewis Pass | *N. solandri* | 265 | 39.2 | 521.2 | 219.4 | 0.355 | 618.7 | 525.9 |
| Lewis Pass | *N. solandri* | 185 | 45.1 | 515.7 | 181.8 | 0.309 | 588.0 | 489.9 |
| Lewis Pass | *N. solandri* | 150 | 48.3 | 510.6 | 198.8 | 0.316 | 628.6 | 602.9 |
| Lewis Pass | *N. solandri* | 188 | 50.0 | 533.3 | 224.0 | 0.333 | 672.3 | 518.2 |
| Lewis Pass | *N. solandri* | 255 | 31.5 | 504.1 | 193.7 | 0.321 | 603.3 | 482.7 |
| Lewis Pass | *N. solandri* | 192 | 29.6 | 525.9 | 206.1 | 0.337 | 610.8 | 582.8 |
| Lewis Pass | *N. solandri* | 273 | 46.5 | 502.9 | 184.9 | 0.339 | 545.2 | 524.5 |
| Lewis Pass | *N. solandri* | 174 | 31.7 | 512.4 | 195.6 | 0.348 | 561.7 | 566.6 |
| Lewis Pass | *N. solandri* | 83 | 47.5 | 505.2 | 204.2 | 0.350 | 583.8 | 512.8 |
| Lewis Pass | *N. solandri* | 105 | 25.9 | 524.3 | 208.5 | 0.348 | 598.4 | 503.8 |
| Lewis Pass | *N. solandri* | 91 | 33.5 | 519.1 | 203.0 | 0.369 | 549.5 | 519.5 |
| Macfarlane Mound | *N. solandri* | 176 | 80.4 | 492.8 | 178.2 | 0.336 | 530.8 | 692.2 |
| Macfarlane Mound | *N. solandri* | 128 | 37.4 | 520.5 | 183.1 | 0.296 | 619.1 | 710.8 |
| Macfarlane Mound | *N. solandri* | 270 | 168.9 | 498.2 | 162.2 | 0.260 | 623.0 | 671.5 |
| Macfarlane Mound | *N. solandri* | 212 | 60.8 | 502.1 | 177.9 | 0.320 | 555.9 | 671.9 |
| Macfarlane Mound | *N. solandri* | 223 | 60.2 | 531.6 | 214.8 | 0.286 | 752.2 | 603.0 |
| Macfarlane Mound | *N. solandri* | 147 | 38.5 | 539.6 | 217.6 | 0.268 | 811.2 | 614.1 |
| Macfarlane Mound | *N. solandri* | 183 | 48.1 | 513.9 | 178.7 | 0.304 | 587.0 | 608.6 |
| Macfarlane Mound | *N. solandri* | 156 | 43.4 | 507.2 | 174.3 | 0.281 | 620.4 | 596.6 |
| Macfarlane Mound | *N. solandri* | 77 | 31.6 | 529.4 | 190.0 | 0.292 | 651.0 | 491.5 |
| Mirza Creek | *N. solandri* | 398 | 53.6 | 526.9 | 164.2 | 0.280 | 585.5 | 661.6 |
| Mirza Creek | *N. solandri* | 518 | 90.1 | 484.7 | 141.0 | 0.261 | 539.2 | 690.1 |
| Mirza Creek | *N. solandri* | 296 | 81.4 | 506.4 | 146.2 | 0.243 | 602.6 | 718.6 |
| Mirza Creek | *N. solandri* | 216 | 108.0 | 485.3 | 152.8 | 0.299 | 510.3 | 617.9 |
| Mirza Creek | *N. solandri* | 440 | 75.5 | 516.5 | 165.6 | 0.268 | 616.9 | 561.0 |
| Mirza Creek | *N. solandri* | 312 | 91.5 | 464.6 | 129.0 | 0.225 | 573.7 | 647.8 |
| Mirza Creek | *N. solandri* | 353 | 100.5 | 493.8 | 158.2 | 0.282 | 561.4 | 595.2 |
| Mirza Creek | *N. solandri* | 502 | 110.6 | 465.5 | 140.1 | 0.254 | 551.3 | 708.1 |
| Mirza Creek | *N. solandri* | 396 | 91.5 | 490.3 | 166.1 | 0.296 | 560.8 | 609.8 |
| Mirza Creek | *N. solandri* | 665 | 90.4 | 477.2 | 150.4 | 0.287 | 524.6 | 630.6 |
| Mt Grey | *N. solandri* | 760 | 98.6 | 490.8 | 134.9 | 0.213 | 632.7 | 657.7 |
| Peasoup Creek | *N. solandri* | 386 | 104.6 | 480.9 | 154.4 | 0.239 | 647.0 | 627.3 |
| Peasoup Creek | *N. solandri* | 475 | 94.2 | 500.0 | 146.5 | 0.279 | 524.7 | 603.9 |
| Peasoup Creek | *N. solandri* | 254 | 50.7 | 480.3 | 144.0 | 0.242 | 595.0 | 581.8 |
| Peasoup Creek | *N. solandri* | 416 | 51.5 | 510.8 | 184.5 | 0.249 | 740.2 | 603.4 |
| Peasoup Creek | *N. solandri* | 379 | 51.1 | 512.0 | 125.2 | 0.247 | 506.7 | 811.5 |
| Peasoup Creek | *N. solandri* | 506 | 62.2 | 513.4 | 160.1 | 0.267 | 600.2 | 611.7 |
| Peasoup Creek | *N. solandri* | 613 | 64.1 | 482.8 | 152.9 | 0.224 | 681.9 | 635.8 |
| Peasoup Creek | *N. solandri* | 652 | 81.8 | 466.1 | 143.0 | 0.202 | 707.4 | 608.8 |
| Piano Flat | *N. solandri* | 251 | 105.8 | 444.4 | 121.0 | 0.272 | 445.1 | 548.8 |
| Piano Flat | *N. solandri* | 267 | 98.7 | 450.8 | 167.2 | 0.396 | 422.6 | 559.7 |
| Piano Flat | *N. solandri* | 508 | 64.9 | 450.8 | 134.1 | 0.268 | 500.6 | 528.4 |
| Piano Flat | *N. solandri* | 346 | 70.6 | 462.8 | 158.6 | 0.349 | 454.8 | 495.9 |
| Piano Flat | *N. solandri* | 216 | 119.8 | 465.4 | 168.6 | 0.377 | 447.5 | 519.2 |
| Piano Flat | *N. solandri* | 381 | 85.9 | 498.4 | 176.9 | 0.362 | 488.3 | 582.3 |
| Piano Flat | *N. solandri* | 387 | 61.9 | 484.2 | 148.6 | 0.335 | 443.4 | 546.5 |
| Piano Flat | *N. solandri* | 410 | 64.6 | 476.2 | 139.3 | 0.332 | 419.9 | 543.8 |
| Piano Flat | *N. solandri* | 71 | 63.9 | 425.4 | 89.2 | 0.224 | 397.5 | 506.8 |
| Piano Flat | *N. solandri* | 95 | 117.3 | 421.6 | 73.3 | 0.204 | 359.0 | 524.8 |
| Tom Creek | *N. solandri* | 646 | 65.2 | 502.2 | 176.4 | 0.346 | 510.4 | 615.5 |
| Tom Creek | *N. solandri* | 130 | 108.9 | 451.5 | 111.1 | 0.234 | 474.4 | 539.5 |
| Tom Creek | *N. solandri* | 250 | 57.5 | 451.4 | 113.0 | 0.246 | 458.8 | 595.1 |
| Tom Creek | *N. solandri* | 309 | 76.2 | 459.2 | 140.4 | 0.303 | 463.1 | 543.2 |
| Tom Creek | *N. solandri* | 300 | 170.2 | 399.3 | 125.7 | 0.316 | 397.4 | 488.9 |
| Tom Creek | *N. solandri* | 336 | 67.3 | 452.3 | 133.7 | 0.269 | 497.5 | 514.2 |
| Tom Creek | *N. solandri* | 310 | 79.6 | 465.2 | 134.4 | 0.281 | 479.1 | 514.7 |
| Tom Creek | *N. solandri* | 174 | 107.7 | 422.9 | 99.4 | 0.235 | 422.8 | 537.4 |
| Tom Creek | *N. solandri* | 118 | 34.9 | 452.4 | 108.9 | 0.238 | 457.5 | 493.8 |
| Tom Creek | *N. solandri* | 69 | 95.8 | 408.1 | 95.0 | 0.229 | 414.1 | 504.6 |
| Tom Creek | *N. solandri* | 85 | 121.7 | 430.1 | 98.6 | 0.231 | 426.1 | 489.3 |
| Woolshed Hill | *N. solandri* | 271 | 84.2 | 517.0 | 180.5 | 0.304 | 593.0 | 576.8 |
| Woolshed Hill | *N. solandri* | 329 | 68.7 | 487.2 | 175.9 | 0.321 | 547.7 | 573.3 |
| Woolshed Hill | *N. solandri* | 332 | 69.9 | 503.3 | 196.4 | 0.331 | 593.6 | 611.2 |
| Woolshed Hill | *N. solandri* | 299 | 67.5 | 481.5 | 140.2 | 0.224 | 625.7 | 525.0 |
| Eglington Valley | *N. fusca* | 351 | 223.8 | 445.0 | 115.7 | 0.239 | 484.6 | 601.5 |
| Eglington Valley | *N. fusca* | 544 | 318.4 | 415.1 | 93.0 | 0.245 | 378.8 | 630.3 |
| Eglington Valley | *N. fusca* | 422 | 361.5 | 415.8 | 88.8 | 0.214 | 414.6 | 639.7 |
| Eglington Valley | *N. fusca* | 300 | 298.4 | 427.7 | 111.9 | 0.248 | 451.3 | 555.7 |
| Eglington Valley | *N. fusca* | 346 | 274.5 | 444.4 | 112.2 | 0.248 | 452.8 | 555.5 |
| Eglington Valley | *N. fusca* | 517 | 265.6 | 426.0 | 98.6 | 0.247 | 399.4 | 621.5 |
| Eglington Valley | *N. fusca* | 573 | 341.8 | 432.1 | 106.2 | 0.266 | 399.6 | 586.3 |
| Karamea Bluff | *N. fusca* | 254 | 346.3 | 375.6 | 72.8 | 0.185 | 392.5 | 529.0 |
| Karamea Bluff | *N. fusca* | 653 | 432.1 | 438.1 | 114.6 | 0.234 | 490.0 | 604.3 |
| Karamea Bluff | *N. fusca* | 380 | 316.3 | 418.7 | 111.6 | 0.234 | 476.1 | 619.4 |
| Karamea Bluff | *N. fusca* | 568 | 440.4 | 425.6 | 116.9 | 0.276 | 423.4 | 631.3 |
| Karamea Bluff | *N. fusca* | 407 | 304.0 | 435.6 | 95.7 | 0.203 | 470.6 | 587.2 |
| Karamea Bluff | *N. fusca* | 330 | 372.8 | 444.8 | 104.0 | 0.208 | 500.8 | 560.1 |
| Karamea Bluff | *N. fusca* | 369 | 379.3 | 408.1 | 92.5 | 0.241 | 384.3 | 553.1 |
| Karamea Bluff | *N. fusca* | 236 | 578.3 | 420.1 | 104.6 | 0.236 | 442.9 | 628.7 |
| Mt Grey | *N. fusca* | 249 | 345.3 | 411.8 | 88.6 | 0.162 | 546.4 | 613.5 |
| Mt Grey | *N. fusca* | 569 | 387.3 | 442.3 | 108.9 | 0.210 | 519.7 | 601.0 |
| Oparara | *N. fusca* | 239 | 488.1 | 447.2 | 108.4 | 0.239 | 453.5 | 572.6 |
| Oparara | *N. fusca* | 191 | 343.6 | 426.0 | 83.8 | 0.203 | 412.9 | 536.8 |
| Oparara | *N. fusca* | 262 | 436.5 | 443.9 | 108.8 | 0.245 | 443.8 | 581.6 |
| Oparara | *N. fusca* | 248 | 367.3 | 456.9 | 111.1 | 0.247 | 449.7 | 514.4 |
| Oparara | *N. fusca* | 341 | 345.0 | 463.2 | 121.7 | 0.246 | 495.7 | 540.0 |
| Oparara | *N. fusca* | 210 | 337.3 | 437.2 | 105.2 | 0.223 | 472.4 | 572.0 |
| Oparara | *N. fusca* | 345 | 377.6 | 431.6 | 96.1 | 0.211 | 456.5 | 429.3 |
| Peasoup Creek | *N. fusca* | 308 | 517.8 | 430.2 | 100.0 | 0.329 | 304.3 | 568.9 |
| Peasoup Creek | *N. fusca* | 338 | 522.5 | 421.1 | 104.1 | 0.287 | 362.1 | 574.7 |
| Peasoup Creek | *N. fusca* | 356 | 379.0 | 472.2 | 109.8 | 0.267 | 411.4 | 592.6 |
| Peasoup Creek | *N. fusca* | 482 | 445.0 | 432.1 | 98.7 | 0.305 | 323.9 | 525.2 |
| Peasoup Creek | *N. fusca* | 498 | 700.1 | 430.1 | 102.4 | 0.312 | 328.7 | 516.3 |
| Peasoup Creek | *N. fusca* | 457 | 576.8 | 430.1 | 112.0 | 0.274 | 408.5 | 545.2 |
| Peasoup Creek | *N. fusca* | 404 | 355.8 | 456.1 | 109.6 | 0.296 | 370.8 | 567.3 |
| Peasoup Creek | *N. fusca* | 184 | 453.8 | 405.6 | 83.3 | 0.287 | 290.4 | 556.8 |
| Piano Flat | *N. fusca* | 508 | 334.4 | 416.6 | 96.3 | 0.229 | 420.9 | 532.8 |
| Piano Flat | *N. fusca* | 257 | 307.1 | 410.8 | 111.7 | 0.252 | 443.2 | 537.0 |
| Piano Flat | *N. fusca* | 283 | 291.4 | 424.4 | 108.8 | 0.253 | 430.7 | 516.4 |
| Piano Flat | *N. fusca* | 655 | 343.8 | 432.1 | 98.0 | 0.218 | 450.1 | 575.7 |
| Piano Flat | *N. fusca* | 398 | 316.7 | 409.8 | 105.5 | 0.247 | 427.0 | 528.1 |
| Piano Flat | *N. fusca* | 555 | 304.2 | 416.0 | 82.2 | 0.193 | 424.9 | 549.1 |
| Piano Flat | *N. fusca* | 377 | 368.1 | 403.4 | 78.2 | 0.205 | 382.4 | 554.0 |
| Piano Flat | *N. fusca* | 410 | 298.5 | 415.8 | 90.1 | 0.213 | 422.7 | 608.8 |
| Staircase Creek | *N. fusca* | 322 | 440.0 | 420.8 | 95.5 | 0.265 | 359.7 | 595.4 |
| Staircase Creek | *N. fusca* | 332 | 433.7 | 411.8 | 79.1 | 0.217 | 364.8 | 565.6 |
| Staircase Creek | *N. fusca* | 214 | 459.0 | 408.1 | 71.4 | 0.201 | 354.9 | 481.9 |
| Staircase Creek | *N. fusca* | 316 | 557.7 | 413.1 | 107.4 | 0.263 | 408.7 | 528.9 |
| Staircase Creek | *N. fusca* | 401 | 472.2 | 400.8 | 82.2 | 0.201 | 408.0 | 509.2 |
| Staircase Creek | *N. fusca* | 174 | 442.0 | 423.2 | 86.0 | 0.215 | 400.2 | 617.7 |
| Staircase Creek | *N. fusca* | 372 | 571.4 | 382.9 | 74.7 | 0.223 | 335.1 | 553.7 |
| Staircase Creek | *N. fusca* | 586 | 703.8 | 391.8 | 84.7 | 0.253 | 334.2 | 561.2 |
| Tom Creek | *N. fusca* | 297 | 362.2 | 410.7 | 85.0 | 0.232 | 366.5 | 584.8 |
| Tom Creek | *N. fusca* | 320 | 355.7 | 396.2 | 88.6 | 0.241 | 367.2 | 578.7 |
| Tom Creek | *N. fusca* | 442 | 426.2 | 370.3 | 72.0 | 0.257 | 280.1 | 547.5 |
| Tom Creek | *N. fusca* | 776 | 199.1 | 431.9 | 138.6 | 0.331 | 419.1 | 608.4 |
| Tom Creek | *N. fusca* | 426 | 462.4 | 395.9 | 83.0 | 0.242 | 343.7 | 533.6 |
| Tom Creek | *N. fusca* | 212 | 307.2 | 418.0 | 90.5 | 0.244 | 371.5 | 553.4 |
| Tom Creek | *N. fusca* | 378 | 330.2 | 415.9 | 93.6 | 0.253 | 369.6 | 518.7 |
| Tom Creek | *N. fusca* | 519 | 360.7 | 437.4 | 119.2 | 0.265 | 450.5 | 539.5 |
| Tom Creek | *N. fusca* | 98 | 356.0 | 378.1 | 71.9 | 0.224 | 320.5 | 554.4 |
| Tom Creek | *N. fusca* | 101 | 399.6 | 387.8 | 81.3 | 0.240 | 338.3 | 512.3 |
| Tom Creek | *N. fusca* | 78 | 159.3 | 410.1 | 97.3 | 0.269 | 361.2 | 487.7 |
| Tom Creek | *N. fusca* | 785 | 203.0 | 386.0 | 84.2 | 0.250 | 336.7 | 592.9 |
| Woolshed Hill | *N. fusca* | 347 | 417.9 | 408.3 | 82.6 | 0.202 | 409.1 | 549.2 |
| Woolshed Hill | *N. fusca* | 716 | 350.5 | 440.6 | 119.5 | 0.241 | 496.9 | 581.0 |
| Woolshed Hill | *N. fusca* | 464 | 400.9 | 423.8 | 92.3 | 0.211 | 437.0 | 574.2 |
| Woolshed Hill | *N. fusca* | 469 | 511.2 | 413.2 | 88.4 | 0.202 | 437.3 | 507.5 |
| Woolshed Hill | *N. fusca* | 316 | 467.1 | 402.8 | 75.1 | 0.184 | 409.3 | 591.0 |
| Mt Burns | *N. menziesii* | 345 | 56.1 | 504.5 | 181.5 | 0.335 | 542.2 | 496.8 |
| Mt Burns | *N. menziesii* | 313 | 49.1 | 519.8 | 187.4 | 0.306 | 612.7 | 491.2 |
| Mt Burns | *N. menziesii* | 230 | 57.5 | 500.0 | 161.1 | 0.304 | 530.8 | 503.8 |
| Mt Burns | *N. menziesii* | 227 | 58.2 | 452.5 | 157.4 | 0.327 | 482.0 | 450.2 |
| Mt Burns | *N. menziesii* | 388 | 51.4 | 524.8 | 187.6 | 0.323 | 581.6 | 511.0 |
| Mt Burns | *N. menziesii* | 378 | 71.4 | 477.6 | 149.0 | 0.285 | 522.6 | 468.5 |
| Charleston forest | *N. menziesii* | 284 | 67.7 | 478.7 | 132.9 | 0.264 | 503.6 | 589.7 |
| Charleston forest | *N. menziesii* | 327 | 69.1 | 498.1 | 186.7 | 0.363 | 514.3 | 523.6 |
| Charleston forest | *N. menziesii* | 191 | 72.0 | 475.1 | 145.8 | 0.297 | 491.0 | 535.1 |
| Charleston forest | *N. menziesii* | 383 | 60.6 | 481.5 | 150.2 | 0.303 | 494.9 | 512.6 |
| Charleston forest | *N. menziesii* | 224 | 75.2 | 460.5 | 131.6 | 0.281 | 468.8 | 581.9 |
| Charleston forest | *N. menziesii* | 390 | 97.8 | 473.1 | 161.6 | 0.353 | 457.7 | 480.9 |
| Charleston forest | *N. menziesii* | 250 | 77.8 | 504.1 | 158.1 | 0.312 | 506.1 | 619.3 |
| Charleston forest | *N. menziesii* | 323 | 55.5 | 502.7 | 165.8 | 0.334 | 496.3 | 547.8 |
| Eglington Valley | *N. menziesii* | 378 | 52.4 | 476.2 | 174.9 | 0.371 | 470.9 | 501.2 |
| Eglington Valley | *N. menziesii* | 233 | 67.6 | 483.6 | 158.6 | 0.334 | 474.3 | 537.3 |
| Eglington Valley | *N. menziesii* | 486 | 69.0 | 508.3 | 178.3 | 0.342 | 520.9 | 566.7 |
| Gertrude Valley | *N. menziesii* | 135 | 53.9 | 486.8 | 170.7 | 0.318 | 536.1 | 482.2 |
| Gertrude Valley | *N. menziesii* | 185 | 87.4 | 477.1 | 148.7 | 0.301 | 494.1 | 504.7 |
| Gertrude Valley | *N. menziesii* | 183 | 73.5 | 492.4 | 201.5 | 0.360 | 560.0 | 548.8 |
| Gertrude Valley | *N. menziesii* | 278 | 60.3 | 500.0 | 164.4 | 0.317 | 519.3 | 514.3 |
| Gertrude Valley | *N. menziesii* | 125 | 65.8 | 491.6 | 161.6 | 0.313 | 516.3 | 529.8 |
| Gertrude Valley | *N. menziesii* | 114 | 61.9 | 478.4 | 163.0 | 0.308 | 529.2 | 515.2 |
| Gertrude Valley | *N. menziesii* | 128 | 71.7 | 491.7 | 166.0 | 0.321 | 517.7 | 530.6 |
| Gertrude Valley | *N. menziesii* | 105 | 60.2 | 493.6 | 173.7 | 0.314 | 553.9 | 503.9 |
| Gertrude Valley | *N. menziesii* | 76 | 79.9 | 489.0 | 185.6 | 0.331 | 561.1 | 489.8 |
| Gertrude Valley | *N. menziesii* | 84 | 58.9 | 465.3 | 145.1 | 0.290 | 500.9 | 449.9 |
| Gertrude Valley | *N. menziesii* | 71 | 71.8 | 493.4 | 156.0 | 0.313 | 498.7 | 480.4 |
| Haast Pass | *N. menziesii* | 262 | 81.7 | 482.1 | 198.3 | 0.381 | 520.4 | 521.1 |
| Haast Pass | *N. menziesii* | 204 | 65.4 | 500.0 | 160.6 | 0.348 | 461.9 | 570.2 |
| Haast Pass | *N. menziesii* | 162 | 44.0 | 487.2 | 172.7 | 0.374 | 462.1 | 552.4 |
| Haast Pass | *N. menziesii* | 491 | 68.8 | 490.1 | 180.2 | 0.382 | 471.6 | 481.5 |
| Haast Pass | *N. menziesii* | 302 | 101.8 | 480.8 | 184.7 | 0.378 | 488.6 | 496.0 |
| Haast Pass | *N. menziesii* | 148 | 84.7 | 475.3 | 204.3 | 0.414 | 492.9 | 547.0 |
| Haast Pass | *N. menziesii* | 321 | 59.4 | 448.1 | 138.0 | 0.329 | 419.9 | 491.5 |
| Haast Pass | *N. menziesii* | 139 | 66.2 | 497.8 | 167.7 | 0.356 | 471.5 | 507.7 |
| Haast Pass | *N. menziesii* | 99 | 78.3 | 516.4 | 181.4 | 0.365 | 497.1 | 476.7 |
| Lake Heron Station | *N. menziesii* | 479 | 59.6 | 469.6 | 139.4 | 0.249 | 559.2 | 473.9 |
| Lake Heron Station | *N. menziesii* | 176 | 58.6 | 480.3 | 138.8 | 0.255 | 543.9 | 601.6 |
| Lake Heron Station | *N. menziesii* | 535 | 59.0 | 532.5 | 185.0 | 0.278 | 666.5 | 457.7 |
| Lake Heron Station | *N. menziesii* | 609 | 69.3 | 496.0 | 136.5 | 0.231 | 591.0 | 490.9 |
| Lake Heron Station | *N. menziesii* | 183 | 90.8 | 481.7 | 133.1 | 0.259 | 514.5 | 568.3 |
| Lake Heron Station | *N. menziesii* | 473 | 50.3 | 497.0 | 163.0 | 0.270 | 604.2 | 537.1 |
| Lake Heron Station | *N. menziesii* | 398 | 61.6 | 510.6 | 162.4 | 0.263 | 617.4 | 516.8 |
| Huxley River | *N. menziesii* | 262 | 52.0 | 508.6 | 171.2 | 0.381 | 449.7 | 522.8 |
| Huxley River | *N. menziesii* | 297 | 64.2 | 495.3 | 163.6 | 0.355 | 460.2 | 534.0 |
| Huxley River | *N. menziesii* | 221 | 81.0 | 488.9 | 163.0 | 0.383 | 425.7 | 524.4 |
| Huxley River | *N. menziesii* | 428 | 101.4 | 478.0 | 171.6 | 0.405 | 423.9 | 515.7 |
| Huxley River | *N. menziesii* | 181 | 86.3 | 496.3 | 155.3 | 0.350 | 443.6 | 464.5 |
| Huxley River | *N. menziesii* | 192 | 80.8 | 492.1 | 154.7 | 0.352 | 439.7 | 550.4 |
| Huxley River | *N. menziesii* | 215 | 59.9 | 491.7 | 180.6 | 0.395 | 457.4 | 556.3 |
| Huxley River | *N. menziesii* | 310 | 77.0 | 498.0 | 162.3 | 0.373 | 435.2 | 529.5 |
| Karamea Bluff | *N. menziesii* | 264 | 77.2 | 422.3 | 112.7 | 0.279 | 403.9 | 581.0 |
| Karamea Bluff | *N. menziesii* | 507 | 49.3 | 507.9 | 196.8 | 0.319 | 616.0 | 545.7 |
| Karamea Bluff | *N. menziesii* | 244 | 60.2 | 476.8 | 187.7 | 0.401 | 468.6 | 660.9 |
| Karamea Bluff | *N. menziesii* | 292 | 67.9 | 474.7 | 151.7 | 0.343 | 442.5 | 532.7 |
| Karamea Bluff | *N. menziesii* | 465 | 89.6 | 472.6 | 154.0 | 0.327 | 471.3 | 583.8 |
| Karamea Bluff | *N. menziesii* | 167 | 105.1 | 464.3 | 123.7 | 0.265 | 467.1 | 562.0 |
| Karamea Bluff | *N. menziesii* | 293 | 78.0 | 483.6 | 132.1 | 0.267 | 494.9 | 610.7 |
| Karamea Bluff | *N. menziesii* | 249 | 89.4 | 469.7 | 130.1 | 0.269 | 482.8 | 580.3 |
| Karamea Bluff | *N. menziesii* | 66 | 86.5 | 446.1 | 105.2 | 0.235 | 447.3 | 534.7 |
| Karamea Bluff | *N. menziesii* | 87 | 110.9 | 471.7 | 112.7 | 0.241 | 468.1 | 545.1 |
| Karamea Bluff | *N. menziesii* | 104 | 131.2 | 421.1 | 109.8 | 0.285 | 384.6 | 521.8 |
| Oparara | *N. menziesii* | 243 | 84.9 | 529.2 | 181.4 | 0.320 | 566.5 | 565.1 |
| Oparara | *N. menziesii* | 162 | 80.6 | 497.7 | 146.2 | 0.281 | 521.1 | 525.4 |
| Oparara | *N. menziesii* | 307 | 80.8 | 495.7 | 156.8 | 0.307 | 510.8 | 538.8 |
| Oparara | *N. menziesii* | 343 | 84.0 | 486.7 | 131.0 | 0.292 | 448.2 | 519.4 |
| Oparara | *N. menziesii* | 340 | 55.1 | 497.2 | 161.5 | 0.303 | 532.7 | 564.5 |
| Oparara | *N. menziesii* | 284 | 83.3 | 472.5 | 123.6 | 0.274 | 451.3 | 527.2 |
| Oparara | *N. menziesii* | 440 | 69.7 | 493.0 | 167.5 | 0.338 | 495.2 | 494.9 |
| Paringa | *N. menziesii* | 209 | 50.1 | 455.9 | 123.8 | 0.276 | 449.0 | 522.0 |
| Paringa | *N. menziesii* | 539 | 85.5 | 456.9 | 142.7 | 0.318 | 448.4 | 559.8 |
| Paringa | *N. menziesii* | 337 | 51.2 | 434.8 | 136.7 | 0.315 | 433.5 | 517.3 |
| Paringa | *N. menziesii* | 572 | 73.0 | 454.5 | 118.3 | 0.268 | 440.8 | 462.3 |
| Paringa | *N. menziesii* | 488 | 81.0 | 481.5 | 144.4 | 0.301 | 479.9 | 521.4 |
| Paringa | *N. menziesii* | 254 | 73.4 | 458.9 | 129.4 | 0.287 | 450.7 | 562.1 |
| Paringa | *N. menziesii* | 85 | 49.6 | 433.7 | 145.2 | 0.363 | 399.7 | 460.5 |
| Paringa | *N. menziesii* | 81 | 51.3 | 440.3 | 115.0 | 0.282 | 407.3 | 449.6 |
| Peasoup Creek | *N. menziesii* | 572 | 56.8 | 538.5 | 172.5 | 0.248 | 695.1 | 566.6 |
| Peasoup Creek | *N. menziesii* | 290 | 85.8 | 528.5 | 162.0 | 0.269 | 601.8 | 682.8 |
| Peasoup Creek | *N. menziesii* | 572 | 69.0 | 538.5 | 177.5 | 0.296 | 600.2 | 554.0 |
| Peasoup Creek | *N. menziesii* | 370 | 70.4 | 548.7 | 176.1 | 0.220 | 801.3 | 591.1 |
| Peasoup Creek | *N. menziesii* | 282 | 54.0 | 532.5 | 166.7 | 0.270 | 618.2 | 606.1 |
| Peasoup Creek | *N. menziesii* | 184 | 36.3 | 530.4 | 168.0 | 0.272 | 616.9 | 502.2 |
| Waikaia slopes | *N. menziesii* | 491 | 60.3 | 474.3 | 137.6 | 0.303 | 454.0 | 478.0 |
| Waikaia slopes | *N. menziesii* | 303 | 66.5 | 485.8 | 180.5 | 0.345 | 522.7 | 464.4 |
| Waikaia slopes | *N. menziesii* | 187 | 71.0 | 445.0 | 119.7 | 0.277 | 431.6 | 437.0 |
| Waikaia slopes | *N. menziesii* | 296 | 63.9 | 489.6 | 147.1 | 0.293 | 501.4 | 541.6 |
| Waikaia slopes | *N. menziesii* | 252 | 55.4 | 490.4 | 139.0 | 0.295 | 471.5 | 467.8 |
| Waikaia slopes | *N. menziesii* | 232 | 60.9 | 481.7 | 151.1 | 0.285 | 530.4 | 557.0 |
| Princhester Hut | *N. menziesii* | 198 | 92.1 | 463.8 | 139.0 | 0.290 | 478.6 | 578.6 |
| Princhester Hut | *N. menziesii* | 227 | 45.1 | 486.3 | 179.4 | 0.359 | 499.8 | 505.9 |
| Princhester Hut | *N. menziesii* | 556 | 95.8 | 470.6 | 158.7 | 0.341 | 465.6 | 496.6 |
| Princhester Hut | *N. menziesii* | 205 | 84.5 | 456.3 | 136.1 | 0.303 | 449.2 | 474.8 |
| Princhester Hut | *N. menziesii* | 180 | 51.1 | 493.3 | 144.8 | 0.309 | 468.0 | 505.3 |
| Princhester Hut | *N. menziesii* | 703 | 60.1 | 471.2 | 163.1 | 0.334 | 488.8 | 502.0 |
| Princhester Hut | *N. menziesii* | 279 | 67.5 | 479.3 | 154.1 | 0.322 | 478.2 | 508.9 |
| Princhester Hut | *N. menziesii* | 281 | 84.9 | 452.5 | 162.5 | 0.354 | 459.2 | 482.0 |
| Roaring Meg | *N. menziesii* | 141 | 91.1 | 488.1 | 143.7 | 0.270 | 531.5 | 461.9 |
| Roaring Meg | *N. menziesii* | 291 | 86.7 | 506.5 | 173.9 | 0.315 | 552.8 | 440.5 |
| Roaring Meg | *N. menziesii* | 361 | 93.7 | 476.5 | 137.7 | 0.288 | 477.6 | 440.7 |
| Roaring Meg | *N. menziesii* | 126 | 93.9 | 488.1 | 153.4 | 0.298 | 514.6 | 492.1 |
| Roaring Meg | *N. menziesii* | 380 | 110.3 | 451.4 | 164.1 | 0.350 | 468.9 | 461.2 |
| Roaring Meg | *N. menziesii* | 283 | 61.8 | 473.7 | 160.2 | 0.318 | 503.1 | 478.7 |
| Roaring Meg | *N. menziesii* | 93 | 102.3 | 454.5 | 151.5 | 0.325 | 466.2 | 472.6 |
| Roaring Meg | *N. menziesii* | 166 | 64.7 | 487.4 | 162.9 | 0.302 | 539.1 | 471.9 |
| Roaring Meg | *N. menziesii* | 93 | 63.5 | 477.2 | 194.6 | 0.344 | 564.9 | 442.9 |
| Roaring Meg | *N. menziesii* | 74 | 89.5 | 459.9 | 151.3 | 0.328 | 460.9 | 459.8 |
| Tom Creek | *N. menziesii* | 156 | 108.8 | 441.2 | 110.3 | 0.298 | 370.6 | 488.4 |
| Tom Creek | *N. menziesii* | 128 | 108.7 | 465.8 | 125.1 | 0.299 | 418.2 | 541.1 |
| Tom Creek | *N. menziesii* | 299 | 112.9 | 496.8 | 138.2 | 0.318 | 435.1 | 481.5 |
| Tom Creek | *N. menziesii* | 278 | 111.0 | 471.9 | 151.4 | 0.341 | 443.3 | 472.1 |
| Tom Creek | *N. menziesii* | 372 | 98.0 | 474.2 | 140.8 | 0.321 | 438.4 | 460.4 |
| Tom Creek | *N. menziesii* | 199 | 107.4 | 462.7 | 144.3 | 0.339 | 425.5 | 535.0 |
| Tom Creek | *N. menziesii* | 207 | 124.8 | 444.9 | 113.2 | 0.282 | 401.2 | 490.8 |
| Tom Creek | *N. menziesii* | 572 | 100.4 | 456.1 | 134.5 | 0.330 | 407.0 | 451.1 |
| Tom Creek | *N. menziesii* | 60 | 132.0 | 393.2 | 96.2 | 0.274 | 350.9 | 454.3 |
| Tom Creek | *N. menziesii* | 113 | 113.0 | 445.3 | 100.9 | 0.276 | 365.8 | 494.5 |
| Tom Creek | *N. menziesii* | 69 | 156.2 | 373.8 | 100.5 | 0.320 | 314.3 | 430.3 |
| Bullock Creek | *N. truncata* | 581 | 227.3 | 467.9 | 134.6 | 0.262 | 513.4 | 690.8 |
| Bullock Creek | *N. truncata* | 601 | 404.7 | 477.6 | 147.5 | 0.294 | 502.1 | 600.2 |
| Bullock Creek | *N. truncata* | 382 | 369.7 | 484.5 | 156.1 | 0.296 | 527.3 | 653.5 |
| Bullock Creek | *N. truncata* | 404 | 558.8 | 455.5 | 139.2 | 0.327 | 425.8 | 650.7 |
| Bullock Creek | *N. truncata* | 384 | 336.4 | 519.8 | 140.6 | 0.255 | 550.5 | 625.5 |
| Bullock Creek | *N. truncata* | 389 | 368.3 | 465.6 | 128.4 | 0.276 | 465.0 | 614.8 |
| Bullock Creek | *N. truncata* | 222 | 315.3 | 460.9 | 129.1 | 0.240 | 537.8 | 654.5 |
| Bullock Creek | *N. truncata* | 111 | 376.6 | 477.9 | 126.1 | 0.257 | 490.4 | 569.3 |
| Bullock Creek | *N. truncata* | 65 | 308.9 | 477.6 | 160.8 | 0.300 | 536.0 | 549.6 |
| Charleston forest | *N. truncata* | 246 | 492.3 | 451.1 | 120.9 | 0.269 | 449.6 | 700.6 |
| Charleston forest | *N. truncata* | 248 | 333.4 | 455.2 | 112.8 | 0.234 | 482.4 | 628.0 |
| Charleston forest | *N. truncata* | 281 | 451.6 | 476.7 | 124.4 | 0.253 | 492.7 | 640.6 |
| Charleston forest | *N. truncata* | 178 | 325.1 | 478.6 | 134.1 | 0.270 | 496.7 | 666.6 |
| Charleston forest | *N. truncata* | 200 | 416.7 | 467.4 | 128.0 | 0.253 | 505.1 | 610.9 |
| Charleston forest | *N. truncata* | 339 | 313.1 | 481.6 | 141.8 | 0.290 | 488.3 | 631.0 |
| Charleston forest | *N. truncata* | 439 | 379.4 | 488.0 | 156.0 | 0.286 | 546.0 | 610.0 |
| Charleston forest | *N. truncata* | 553 | 396.0 | 476.9 | 137.9 | 0.279 | 494.5 | 631.8 |
| Granville | *N. truncata* | 749 | 385.3 | 448.6 | 131.3 | 0.299 | 439.8 | 577.8 |
| Granville | *N. truncata* | 308 | 249.7 | 481.8 | 132.2 | 0.269 | 491.7 | 641.4 |
| Granville | *N. truncata* | 189 | 193.1 | 484.8 | 132.1 | 0.234 | 563.9 | 663.8 |
| Granville | *N. truncata* | 603 | 287.3 | 443.4 | 117.3 | 0.249 | 471.1 | 661.7 |
| Granville | *N. truncata* | 164 | 218.8 | 487.4 | 150.4 | 0.263 | 571.3 | 670.5 |
| Granville | *N. truncata* | 309 | 163.7 | 484.6 | 124.6 | 0.236 | 528.5 | 657.4 |
| Granville | *N. truncata* | 230 | 184.7 | 483.9 | 122.4 | 0.231 | 530.2 | 640.0 |
| Granville | *N. truncata* | 307 | 247.7 | 500.0 | 157.4 | 0.309 | 509.9 | 672.9 |
| Lake Hanlon | *N. truncata* | 182 | 397.1 | 478.1 | 158.3 | 0.288 | 549.0 | 669.3 |
| Lake Hanlon | *N. truncata* | 266 | 354.7 | 486.6 | 163.8 | 0.301 | 544.2 | 675.0 |
| Lake Hanlon | *N. truncata* | 192 | 345.4 | 481.4 | 149.8 | 0.293 | 510.7 | 644.5 |
| Lake Hanlon | *N. truncata* | 546 | 355.1 | 509.4 | 167.3 | 0.295 | 567.4 | 656.1 |
| Lake Hanlon | *N. truncata* | 480 | 340.3 | 488.2 | 166.8 | 0.328 | 508.5 | 643.2 |
| Lake Hanlon | *N. truncata* | 275 | 380.0 | 514.0 | 153.1 | 0.298 | 514.3 | 603.0 |
| Lake Hanlon | *N. truncata* | 382 | 342.1 | 490.4 | 141.5 | 0.294 | 481.5 | 673.9 |
| Lake Hanlon | *N. truncata* | 491 | 286.4 | 486.3 | 161.7 | 0.290 | 557.5 | 637.2 |
| Karamea Bluff | *N. truncata* | 566 | 342.3 | 469.8 | 125.0 | 0.255 | 490.3 | 609.5 |
| Karamea Bluff | *N. truncata* | 393 | 317.7 | 458.9 | 125.9 | 0.266 | 473.4 | 687.0 |
| Karamea Bluff | *N. truncata* | 346 | 364.0 | 440.1 | 118.1 | 0.261 | 451.9 | 592.3 |
| Karamea Bluff | *N. truncata* | 177 | 333.3 | 400.6 | 85.2 | 0.195 | 437.9 | 682.7 |
| Karamea Bluff | *N. truncata* | 478 | 453.6 | 444.8 | 118.2 | 0.257 | 459.1 | 698.4 |
| Karamea Bluff | *N. truncata* | 271 | 351.8 | 455.6 | 128.5 | 0.295 | 436.1 | 666.4 |
| Karamea Bluff | *N. truncata* | 184 | 450.1 | 450.2 | 115.5 | 0.259 | 445.7 | 577.7 |
| Karamea Bluff | *N. truncata* | 394 | 247.9 | 466.1 | 130.8 | 0.268 | 488.2 | 650.5 |
| Karamea Bluff | *N. truncata* | 114 | 334.4 | 458.4 | 141.7 | 0.283 | 500.9 | 597.7 |
| Macfarlane Mound | *N. truncata* | 284 | 325.4 | 477.6 | 155.7 | 0.233 | 669.4 | 729.5 |
| Macfarlane Mound | *N. truncata* | 248 | 349.6 | 475.3 | 143.3 | 0.234 | 613.5 | 680.6 |
| Macfarlane Mound | *N. truncata* | 265 | 245.9 | 489.7 | 187.0 | 0.286 | 653.6 | 775.6 |
| Macfarlane Mound | *N. truncata* | 218 | 191.1 | 480.2 | 148.3 | 0.241 | 615.3 | 639.0 |
| Macfarlane Mound | *N. truncata* | 390 | 183.7 | 493.5 | 143.7 | 0.264 | 544.0 | 701.8 |
| Macfarlane Mound | *N. truncata* | 188 | 397.3 | 486.6 | 173.4 | 0.230 | 755.3 | 755.9 |
| Macfarlane Mound | *N. truncata* | 78 | 280.6 | 462.1 | 121.5 | 0.225 | 539.4 | 657.1 |
| Macfarlane Mound | *N. truncata* | 83 | 167.9 | 479.9 | 142.3 | 0.229 | 622.1 | 645.7 |
| Peasoup Creek | *N. truncata* | 317 | 199.2 | 510.0 | 166.7 | 0.307 | 542.2 | 623.5 |
| Peasoup Creek | *N. truncata* | 426 | 354.5 | 480.8 | 151.5 | 0.281 | 539.8 | 650.8 |
| Peasoup Creek | *N. truncata* | 346 | 336.8 | 480.5 | 124.1 | 0.256 | 484.8 | 593.8 |
| Peasoup Creek | *N. truncata* | 442 | 326.4 | 482.6 | 131.4 | 0.260 | 505.1 | 545.9 |
| Peasoup Creek | *N. truncata* | 339 | 401.6 | 452.2 | 107.1 | 0.242 | 443.2 | 640.2 |
| Peasoup Creek | *N. truncata* | 162 | 326.8 | 459.3 | 110.5 | 0.242 | 456.8 | 617.1 |
| Ronga Saddle | *N. truncata* | 386 | 403.7 | 445.9 | 114.4 | 0.266 | 429.6 | 630.9 |
| Ronga Saddle | *N. truncata* | 454 | 437.1 | 473.8 | 103.6 | 0.213 | 486.6 | 605.5 |
| Ronga Saddle | *N. truncata* | 309 | 339.1 | 490.4 | 174.0 | 0.285 | 611.3 | 586.3 |
| Ronga Saddle | *N. truncata* | 337 | 451.0 | 462.5 | 136.8 | 0.295 | 463.4 | 611.7 |
| Ronga Saddle | *N. truncata* | 242 | 379.1 | 485.1 | 137.4 | 0.285 | 482.6 | 656.0 |
| Ronga Saddle | *N. truncata* | 220 | 432.7 | 479.7 | 119.9 | 0.241 | 496.9 | 660.4 |
| Ronga Saddle | *N. truncata* | 340 | 357.9 | 478.6 | 124.9 | 0.252 | 495.2 | 630.4 |
| Ronga Saddle | *N. truncata* | 248 | 360.5 | 481.2 | 159.5 | 0.275 | 580.0 | 670.0 |
| Ronga Saddle | *N. truncata* | 105 | 344.7 | 479.5 | 145.9 | 0.263 | 555.7 | 614.7 |
